# Calpains Mediate Integrin Attachment Complex Maintenance of Adult Muscle in *Caenorhabditis elegans*

**DOI:** 10.1371/journal.pgen.1002471

**Published:** 2012-01-12

**Authors:** Timothy Etheridge, Elizabeth A. Oczypok, Susann Lehmann, Brandon D. Fields, Freya Shephard, Lewis A. Jacobson, Nathaniel J. Szewczyk

**Affiliations:** 1 School of Graduate Entry Medicine and Health, University of Nottingham, Royal Derby Hospital, Derby, United Kingdom; 2 Department of Biological Sciences, University of Pittsburgh, Pittsburgh, Pennsylvania, United States of America; The Jackson Laboratory, United States of America

## Abstract

Two components of integrin containing attachment complexes, UNC-97/PINCH and UNC-112/MIG-2/Kindlin-2, were recently identified as negative regulators of muscle protein degradation and as having decreased mRNA levels in response to spaceflight. Integrin complexes transmit force between the inside and outside of muscle cells and signal changes in muscle size in response to force and, perhaps, disuse. We therefore investigated the effects of acute decreases in expression of the genes encoding these multi-protein complexes. We find that in fully developed adult *Caenorhabditis elegans* muscle, RNAi against genes encoding core, and peripheral, members of these complexes induces protein degradation, myofibrillar and mitochondrial dystrophies, and a movement defect. Genetic disruption of Z-line– or M-line–specific complex members is sufficient to induce these defects. We confirmed that defects occur in temperature-sensitive mutants for two of the genes: *unc-52*, which encodes the extra-cellular ligand Perlecan, and *unc-112*, which encodes the intracellular component Kindlin-2. These results demonstrate that integrin containing attachment complexes, as a whole, are required for proper maintenance of adult muscle. These defects, and collapse of arrayed attachment complexes into ball like structures, are blocked when DIM-1 levels are reduced. Degradation is also blocked by RNAi or drugs targeting calpains, implying that disruption of integrin containing complexes results in calpain activation. In wild-type animals, either during development or in adults, RNAi against calpain genes results in integrin muscle attachment disruptions and consequent sub-cellular defects. These results demonstrate that calpains are required for proper assembly and maintenance of integrin attachment complexes. Taken together our data provide *in vivo* evidence that a calpain-based molecular repair mechanism exists for dealing with attachment complex disruption in adult muscle. Since *C. elegans* lacks satellite cells, this mechanism is intrinsic to the muscles and raises the question if such a mechanism also exists in higher metazoans.

## Author Summary

Muscle is a dynamic tissue that grows in response to use and nutrition and shrinks in response to lack of use, poor nutrition, or disease. Loss of muscle mass is an important public health problem, but we understand little of the genes that regulate muscle shrinkage. We have found that, in adult worm muscle, attachment to the basement membrane is continuously required to prevent catastrophic sub-cellular defects that result in impaired ability of muscle to function. We have also identified a group of proteases that are activated when the attachment fails to be properly maintained. Conversely, when these proteases are lacking in adult muscle, the muscles fail to maintain attachment to the basement membrane. Thus, we have discovered a group of proteases that appear to act to maintain attachment to the basement membrane and therefore to maintain muscle itself. Because these worms lack satellite cells, this maintenance system is intrinsic to muscle, thus raising the question whether a similar or identical system also works in humans.

## Introduction

Muscle is a multifunctional tissue [1]–[4] with a well appreciated role in locomotion. The contractile properties of muscle that allow for coordinated locomotion require a complex protein based machinery [5] and substantial metabolic input [6]. To balance demand with metabolic cost, the quantity of muscle protein is controlled by both use and nutrition. The regulation of muscle protein content is an area of broad interest owing to the fact that locomotion is an essential part of being human, the general acceptance that muscle is important for athletic prowess, and because specific muscle wasting is a clinical problem. These wasting conditions have substantial negative impact on mortality [7], [8], morbidity, and public health expenditure [9], [10].

Conceptually, muscle size is controlled by signals that regulate the balance of muscle protein synthesis and degradation. When bulk protein synthesis exceeds bulk degradation, growth can occur and when bulk protein degradation exceeds bulk synthesis atrophy occurs. While there are a number of ways in which a net shift in balance can lead to atrophy (e.g. protein synthesis and degradation can each go up or down together or independently and/or to different degrees), degradation is required for atrophy to occur. Four main proteolytic systems, the proteasomes [11], [12], lysosomes [13], calpains [14], and caspases [15], have been identified as key players in the regulation of muscle size and function. However, despite our knowledge of these proteases we know relatively little of how their activities are regulated by the vast array of extra-muscular signals which appear to control muscle size [16].

Our laboratories have developed the soil nematode *Caenorhabditis elegans*, a validated muscle and systems biology model, into a model for the discovery of regulatory signals of muscle protein degradation. As with mammalian muscle, protein degradation in *C. elegans* is observed in response to starvation [17], denervation [18], or disruption of endocrine signalling [19], [20]. Motor neurons release acetylcholine, which acts to inhibit proteasome based degradation in post-synaptic muscle. When animals are starved or “genetically denervated,” proteasome based degradation occurs unless the animals are supplemented with cholinergic agonist [17], [18]. Additionally, muscle itself releases Fibroblast Growth Factor [21] which acts to activate autophagic degradation [19]. This constitutive degradation is prevented when Insulin/Insulin-like Growth Factor, from an unknown source, counterbalances the Fibroblast Growth Factor signalling within muscle [20]. Thus, we have begun to gain a picture of the integrated control of muscle protein degradation in *C. elegans* muscle. Open questions include how calpains and caspases are regulated by extra-muscular signals and how many intra-muscular signalling networks control these four proteolytic systems.

Recently it was shown that gene expression in *C. elegans* muscles responds similarly to mammalian muscle gene expression during spaceflight, with several key genes (for example, MyoD and myosin heavy chain) showing similar changes [22]. Two of the roughly 150 muscle genes which were identified as being down regulated in response to spaceflight, *unc-97* [23] and *unc-112* [24], produce proteins that are part of integrin containing muscle attachment complexes. Integrin-based attachment complexes are essential for proper muscle development [25], show changes in protein content in response to loading and unloading [26], modulate load induced changes in muscle protein synthesis [27], and serve various other essential cellular functions (reviewed in [28]). A recent genomic screen also uncovered *unc-97* and *unc-112* as negative regulators of muscle protein degradation [29]. These observations prompted us to investigate if these attachment complexes, as a whole, functioned as negative regulators of muscle protein degradation in fully differentiated muscle. To do this, we used RNAi to knock down the gene products of the core complex components [28], [30]–[32]: the extracellular ligand, UNC-52/Perlecan; the receptor, PAT-2/Integrin alpha and PAT-3/Integrin beta; and intracellular partners, found at both the Z and M-lines [31], [32], PAT-4/Integrin linked kinase, PAT-6/Actopaxin, UNC-112/MIG-2/Kindlin-2, and UNC-97/PINCH. We also used RNAi to knock down a sub-set of gene products that are peripheral components of the complex. For this we chose: the Z and M-line proteins TLN-1/Talin and ZYX-1/Zyxin [31]–[33]; the Z-line specific proteins ATN-1/alpha actinin and DEB-1/Vinculin [31], [32]; and the M-line specific proteins UNC-82 and UNC-89/Obscurin [31], [32], [34], [35]. As an alternative hypothesis to the complexes as a whole regulating muscle protein degradation, we also tested a known binding partner of UNC-112, UIG-1 [36] and the Rho GTPase, CDC-42, for which UIG-1 is a guanine nucleotide exchange factor [36].

Here we report that integrin attachment complexes are required for proper maintenance of adult muscle and that failure to maintain these complexes results in activation of calpain proteases, general degradation of soluble muscle proteins, myofibrillar and mitochondrial dystrophies, and a severe movement defect. Because the integrin attachment complex member DEB-1/Vinculin is degraded by these activated calpains, we postulate that calpain activation in response to disruption of integrin attachment complexes allows for the reassembly and/or repair of these complexes. In normal adult *C. elegans*, complex disruption may occur as the result of increased mechanical strain and/or failure of other mechanisms to properly coordinate growth of muscle and adjacent hypodermal cells. Thus, calpains help adult muscle maintain both structural integrity and cross tissue communication.

## Results

### Acute genetic disruption of either Z- or M-line muscle attachment complexes results in protease activation

**Figure 1:**
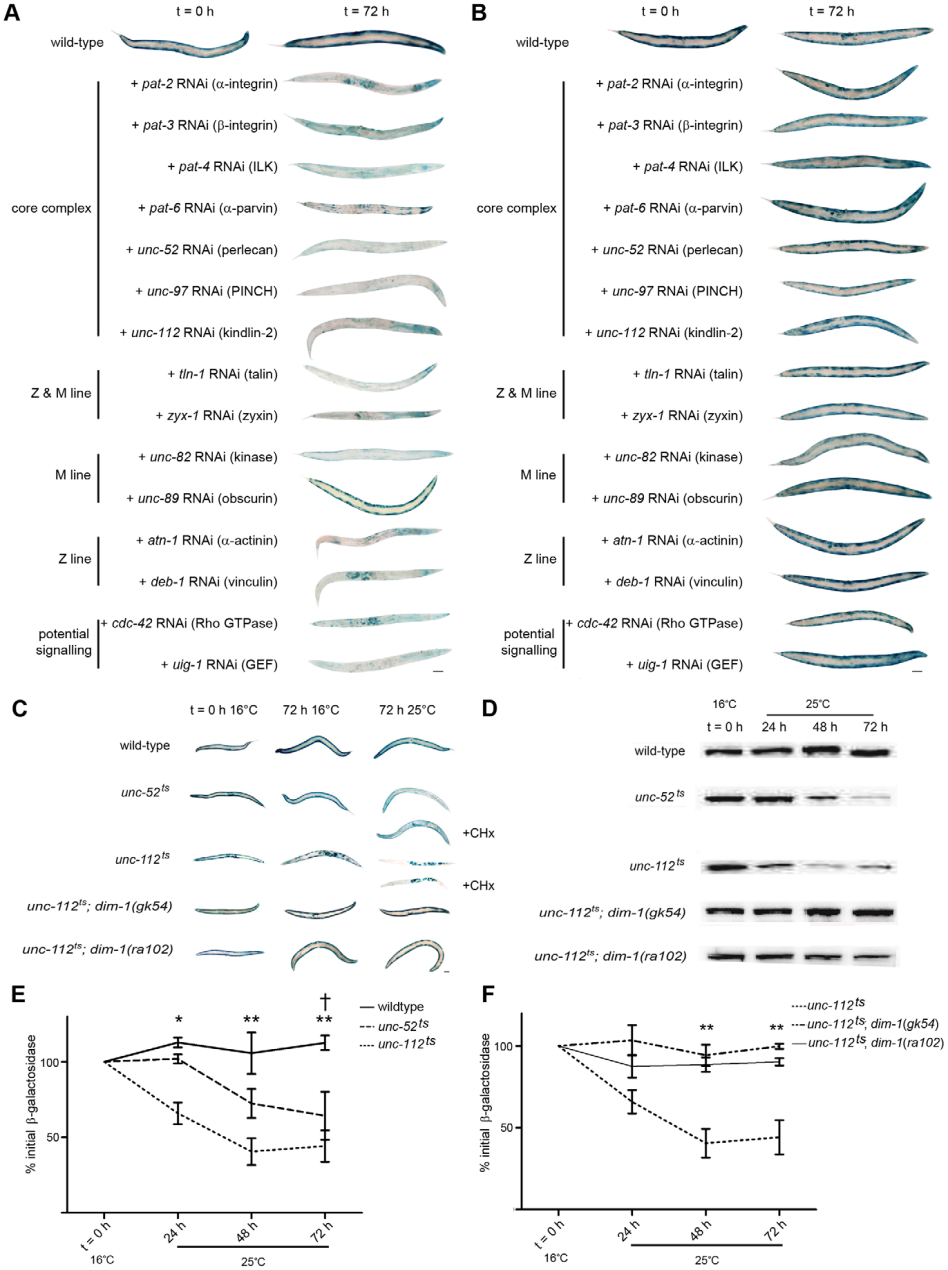
Acute loss of integrin-based attachment induces general cytosolic protein degradation via a common mechanism. A) Age synchronised wild-type L1 larvae were grown to young adulthood at 16°C (t = 0 h) before transferring to NGM RNAi plates [87] seeded with bacteria expressing dsRNA against genes indicated for an additional 72 h (mid-adulthood) at 20°C. The blue stain that appears as circles in the centre of t = 72 h animals is stain in muscles of developing embryos (for example+*pat-2* and *deb-1* RNAi). The blue stain that appears as lines in the t = 72 h animals is indicative of *lacZ* expressing bacteria in the gut (typically near the head, for example+*unc-112* RNAi). B) *dim-1(ra102)* mutants were cultured identically to A. C) Wild-type, *unc-52^ts^*, *unc-112^ts^*, *unc-112^ts^*; *dim-1(gk54)*, and *unc-112^ts^*; *dim-1(ra102)* animals were age synchronised at L1 stage and grown to young adulthood (t = 0 h) at 16°C, and cultured for an additional 72 h at either 16°C (permissive temperature for the mutation) or 25°C (non-permissive temperature). *unc-52^ts^* and *unc-112^ts^* animals were also cultured under the same conditions in the presence of cycloheximide (+CHx) at 400 µg/ml. In A, B and C approximately 20–30 animals were stained for β-galactosidase activity (blue) at t = 0 h and after 24 h, 48 h (not shown) and 72 h. D) Representative immunoblot analysis of 146-kDa β-galactosidase fusion protein in 30-worm lysates, cultured under the same conditions as in C after temperature-shift to 25°C only. All experiments in A, B, C and D were repeated a minimum of three times. E) Kinetics of loss of β-galactosidase protein from 16°C (t = 0 h) after temperature-shift to 25°C in wild-type (solid line), *unc-52^ts^* (large dashed line) or *unc-112^ts^* (small dashed line) animals. *,**Significant difference between *unc-112^ts^* versus wild-type (P<0.01, P<0.001). †Significant difference between *unc-52^ts^* versus wild-type (P<0.01). F) Kinetics of loss of β-galactosidase protein from 16°C (t = 0 h) after temperature-shift to 25°C in *unc-112^ts^* (small dashed line), *unc-112^ts^*; *dim-1(gk54)* (large dashed line) or *unc-112* ^ts^; *dim-1(ra102)* (solid line) animals. **Significant difference between *unc-112^ts^* versus *unc-112^ts^*; *dim-1(gk54)* and *unc-112^ts^*; *dim-1(ra102)* (P<0.001). Values in E and F are the average of three immunoblots ± SEM. Level of significance in all indicated cases from two way repeated measures ANOVA. Scale bars represent 100 µm.

In fully developed adult worms, acute RNAi treatment against any one of fourteen genes that encode integrin muscle attachment complex components resulted in loss of a transgene-encoded LacZ reporter of muscle protein degradation in the cytosol (Figure 1A). Because this reporter protein is synthesised only until adulthood [17] and remains stable for the next 72 to 96 hours in well-fed wild-type animals [18]–[20], [37], [38], loss of reporter indicates that proteases have been activated and degradation is occurring. As RNAi against the core complex components PAT-2, PAT-3, PAT-4, PAT-6, UNC-52, UNC-97, and UNC-112 all yielded protein degradation, it appears that protease activation occurs in response to disruption of the core integrin complex. Consistent with this, RNAi against peripheral components located at both the Z and M-line (TLN-1 and ZYX-1), only the Z-line (ATN-1 and DEB-1), or only the M-line (UNC-82) results in protein degradation. Thus, genetic disruption of either Z-line or M-line specific components is sufficient to result in protease activation. These results suggest that sustained disruption of any integrin containing complex results in activation of a protease in adult *C. elegans* muscle.

**Figure 2:**
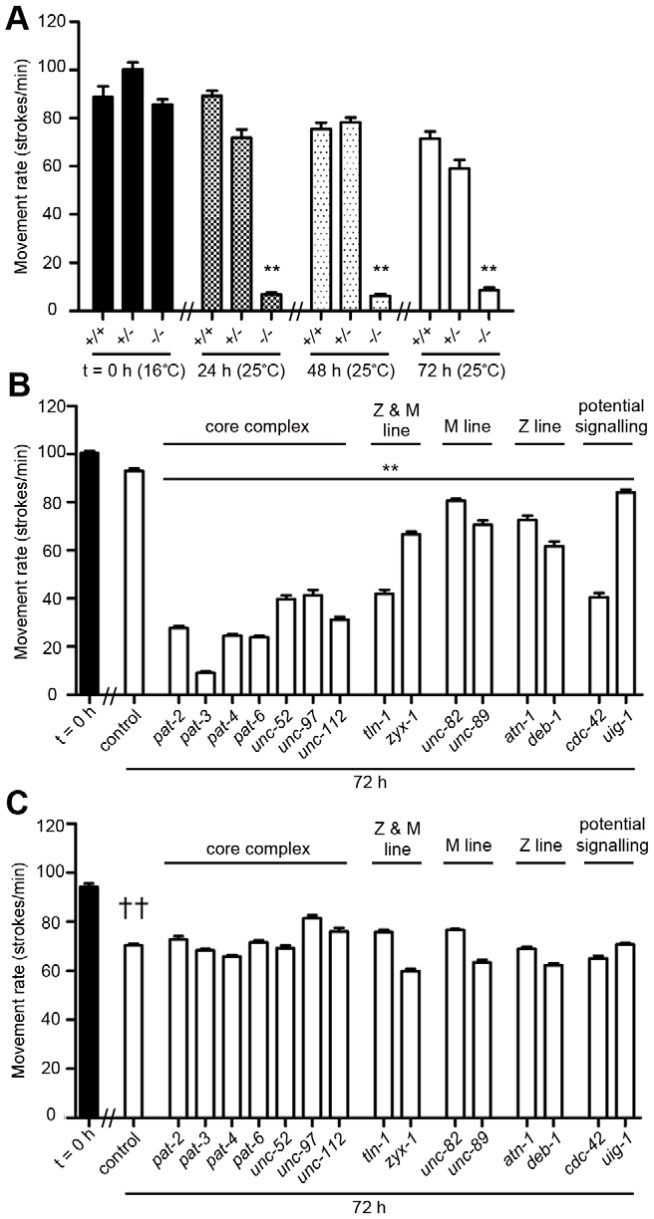
Acute loss of muscle attachment results in a movement defect that does not occur in *dim-1(ra102)* mutants. A) Age synchronised wild-type (+/+), *unc-112^ts^* heterozygotes (+/−) and *unc-112^ts^* homozygotes (−/−) were grown to young adulthood at 16°C (t = 0 h) and cultured after temperature-shift to 25°C (mutation active temperature) for an additional 72 h. At t = 0 h, 24 h, 48 h and 72 h animals were analysed for movement rate by placing animals in BU buffer and counting the number of sinusoidal movements completed in one minute. Values shown are the average ± SEM of 5 animals measured 10 times to give a total of 50 independent measurements. **Significant movement decrease versus +/+ and +/− at respective time point (P<0.001). B) Wild-type and C) *dim-1(ra102)* animals were grown to young adulthood and cultured on RNAi plates [87] against genes indicated on x-axis for an additional 72 h. Movement rate was measured as in A at t = 0 h and 72 h. Values shown are the average ± SEM of 10 animals measured 10 times to give a total of 100 independent measurements. **Significant movement decrease on respective RNAi treatment at 72 h versus wild-type control (P<0.001). ††Significant movement decrease in *dim-1(ra102)* mutants versus wild-type controls at 72 h (P<0.001). All experiments were repeated at least twice. All significance values are from two way repeated measures ANOVA.

**Figure 3:**
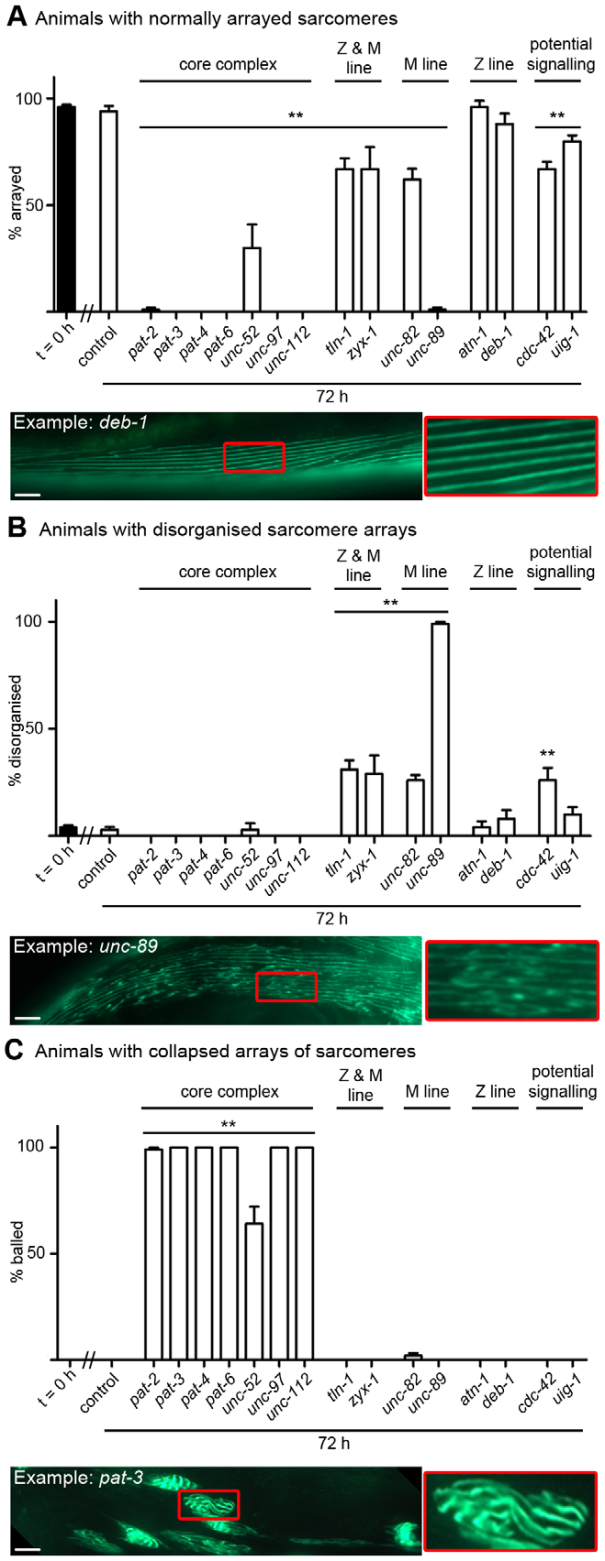
Acute loss of muscle attachment causes disorganisation and collapse of arrayed sarcomeres. Animals expressing a full length translational fusion of *gfp* to *myo-3* (myosin heavy chain A) were age synchronised at L1 stage and grown to young adulthood at 16°C (t = 0 h). Adult animals were then transferred to NGM RNAi plates [87] seeded with bacteria expressing dsRNA against genes indicated for a further 72 h to mid-adulthood. The 20 most Unc animals were picked and scored for identical defects in sarcomere structure in at least two muscles within the animal and this was repeated for 5 independent RNAi treatments (n = 100 animals per condition/time point). A) Percentage of animals where only normal arrays of sarcomeres were observed is displayed as average ± SEM. Below the graph is an example of an RNAi treated animal displaying normal arrays of sarcomeres (as indicated by straight parallel lines of GFP), these are enlarged 300% to the right of the micrograph. B) Percentage of animals where disorganisation of sarcomere arrays were observed is displayed as average ± SEM. Below the graph is an example of an RNAi treated animal displaying disorganised arrays of sarcomeres (as indicated by lack of straight parallel lines of GFP), these are enlarged 300% to the right of the micrograph. C) Percentage of animals where sarcomere arrays have collapsed into ball like structures is displayed as average ± SEM. Below the graph is an example of an RNAi treated animal displaying a collapsed array of sarcomeres (as indicated by parallel lines of GFP that are not straight, long, lines), these are enlarged 300% to the right of the micrograph. A sample image of an RNAi treatment against each gene can be found in Figure S1, not displayed here are animals with tears in the arrayed sarcomeres (see *unc-52* in Figure S1). **Significant difference from control t = 72 h, P<0.001 (two way repeated measures ANOVA). Scale bars represent 15 µm.

RNAi knock down of one of the fifteen genes tested, *unc-89*, a known M-line attachment complex component [39], did not provoke degradation of our transgenic reporter. Below, we report that RNAi against UNC-89 does yield a movement defect and also disruption of the normally arrayed sarcomeres in 100% of animals examined (see below, Figure 2 and Figure 3). Thus, the lack of degradation is not simply due to lack of an effective RNAi treatment. From these results we tentatively conclude that reduced amounts of UNC-89 are not sufficient to cause sustained protease activation. However, additional studies are required to (dis)prove the role of reduced levels of UNC-89 with respect to sustained, or transient, protease activation. It may be that the unique pattern of sub-cellular pathologies seen in response to *unc-89* RNAi treatment (see below) coupled with the lack of observed degradation of LacZ suggests that UNC-89 acts as a key molecule in the intramuscular maintenance of the normal arraying of attachment complexes.

### Sustained disruption of muscle attachment complexes results in general degradation of soluble proteins

Past studies have shown that when the transgene-encoded LacZ reporter utilised here is degraded, so are other transgene-coded proteins expressed in muscle and so are endogenous cytosolic muscle proteins such as arginine kinase (the worm equivalent of creatine kinase) [17], [19], [20], [37], [38]. Thus, the LacZ protein reports on general rather than protein-specific degradation in the muscle cytosol. To confirm that the degradation observed in response to genetic disruption of the integrin complexes was not specific for LacZ, we treated animals containing cytsosolic Green Fluorescent Protein (GFP) or cytosolic DsRed reporters with RNAi against *unc-97* or *unc-112* (the other genes in Figure 1A were not tested). As expected, GFP and DsRed were also degraded (not shown). We did not observe GFP or DsRed leaking out of the muscle during RNAi treatment (not shown), further confirming that the decrease in reporter protein content is the result of intramuscular degradation rather than loss of cytosol due to impaired membrane maintenance.

As RNAi is a relatively new technology, we also confirmed the RNAi results utilizing mutants. We tested temperature sensitive mutants in two members of this attachment complex, UNC-52/Perlecan (an integrin ligand in the basement membrane) [40] and UNC-112/Kindlin-2 (an intramuscular binding partner of the integrin receptor) [24]. As shown in Figure 1C, acute temperature shift of fully developed adult animals results in protein degradation in both *unc-52^ts^* and *unc-112^ts^* mutants but not in wild-type animals. Degradation of the pre-existing LacZ reporter was confirmed by western blot analysis (Figure 1D and 1E). Degradation was not observed in *unc-112^ts^* animals when *unc-112^+^::GFP* was also present (not shown). Whole body protein, as assessed in triplicate 30 worm samples by coomassie staining and quantified in ImageJ, was reduced in *unc-112^ts^* mutants, but not wild-type animals, 48 hours post temperature shift (17%+/−8% loss vs. 40%+/−9% gain, P<0.001 two way repeated measures ANOVA). The decline in total protein in *unc-112^ts^* mutants further supports the inference that sustained genetic disruption of integrin attachment complexes results in sustained activation of a protease, which results in general degradation of soluble muscle protein.

RNAi knockdown reduces the amount of normal gene product whereas the temperature sensitive mutants produce proteins that are structurally and functionally abnormal. Therefore, the similarity of muscle phenotypes (more are reported below) suggests that the trigger for these phenotypes is the reduction of attachment complex function, rather than just aberrant assembly during RNAi knockdown. We believe that a severe reduction of function is required, because we found that *unc-112^ts^/+*heterozygotes at 25°C did not degrade LacZ reporter and showed no signs of sarcomere disorganisation (not shown). Additionally, degradation was prevented in *unc-112^ts^; unc-112^+^::GFP* animals that had low enough levels of GFP to be undetectable on our epifluorescent microscope (not shown). Thus, the observed degradation of cytosolic protein content may represent the consequence of catastrophic failure of the attachment complexes and/or sustained inability to reassemble partially functional complexes.

### Protein degradation in response to muscle attachment complex disruption does not require synthesis of proteases

We next asked whether the reporter degradation was carried out by activation of proteases newly synthesized after disruption of the attachment complexes, or by activation of pre-existing protease(s). We conducted the same temperature shift experiments described above in the presence of the protein synthesis inhibitor cycloheximide (CHx) and found that degradation was indeed occurring (Figure 1C: *unc-52^ts^* or *unc-112^ts^*+CHx). This result suggests that pre-existing proteases are sufficient to account for the protein degradation observed in response to disruption of the integrin complexes.

### *dim-1* mutants do not display protein degradation in response to acute RNAi treatment targeting core attachment complex members

The fact that the gene products (Figure 1A) occur together in attachment complexes does not uniquely establish that a common mechanism is responsible for the common catabolic response to knockdown of any of these proteins. We therefore asked if the response to the various knockdowns could be suppressed by mutation in a single gene. In a screen for second-site mutations that suppress the movement defect of *unc-112* mutants, only mutations in *dim-1* were recovered and characterized [41]. *dim-1* encodes a novel immunoglobulin-like repeat protein that localizes around and between, but not within, Z-lines [42]. RNAi knockdown of any attachment complex gene in a *dim-1* mutant background showed no obvious LacZ degradation (Figure 1B). Temperature shift experiments on *unc-112^ts^* mutants in a *dim-1* mutant background (Figure 1C) also did not show obvious degradation and western blot analysis confirmed that degradation was suppressed (Figure 1D and 1F). Thus, the mutant studies confirm the RNAi results, suggesting that the protein degradation induced by impaired muscle attachment acts via a common mechanism, regardless of which component of the attachment complex is knocked down. Our data indicate that integrin based attachments are not only important for proper development of muscle, they are also essential to maintenance of cytosolic muscle protein content.

### Acute genetic disruption of muscle attachment complexes results in a movement defect in wild-type but not *dim-1* mutants

To determine whether attachment complex disruption had additional effects on adult muscle cells, we next examined gross movement. Disruption of integrin-based attachment complexes by mutation (Figure 2A) or RNAi knockdown of any one of the attachment complex genes tested (Figure 2B) also leads to a decline in animal mobility. Although the *unc-112^ts^* mutants are not fully normal in movement rate even when grown at the “permissive” 16°C, they show a pronounced loss of mobility within 24 h after a shift to 25°C. This does not occur in *unc-112^ts^/+*heterozygotes (Figure 2A) or *unc-112^ts^; unc-112^+^::GFP* animals where the GFP is not visible (not shown). This implies that the attachment complexes function at least mostly normally when wild-type and mutant UNC-112 molecules, which presumably mix randomly during attachment complex assembly, are present. This also implies that the phenotypes of *unc-112^ts^* mutants likely derive from a reduction in the function of pre-formed attachment complexes upon an increase in temperature, and that reduction of UNC-112 function to 50% of normal (the presumed situation in a heterozygote) is not sufficient to disrupt function of the attachment complexes. The functional consequences of RNAi knockdowns in adult animals (Figure 2B) must be understood in this light and further suggest a dynamic state of the attachment complexes in adult animals.

The largest declines in movement following 72 hour treatment with RNAi were observed for the treatments targeting core integrin complex members (PAT-2, PAT-3, PAT-4, PAT-6, UNC-52, UNC-97, UNC-112). These declines are significantly different than for all more peripheral complex members other than CDC-42 and TLN-1. This suggests that genetic disruption of the core complex members, which are found at all attachment sites, has more severe functional consequences than disruption of the more peripheral and/or Z or M-line specific components. However, caution should be applied when analysing quantitative differences between defects seen in response to non-quantitative genetic disruption of multi-protein complexes for which the *in vivo* stoichiometries and protein to protein binding affinities are not known.

We also tested if the movement defect was suppressed in *dim-1* mutants. These mutants, without RNAi treatments, move somewhat more slowly than wild-type animals, yet do not show further depression of movement upon RNAi knockdown of many of the genes whose knockdown cause the greatest movement impairments in wild-type (for example the core complex, compare Figure 2B and 2C). This finding is in line with previous reports that impaired basal movement can prevent functional decline in muscular dystrophy gene mutants [43], [44].

### Acute genetic disruption of muscle attachment complexes results in disorganisation and collapse of arrayed sarcomeres

Acute RNAi treatment targeting each of the muscle attachment complex genes tested causes myofibrillar defects (Figure 3, Figure S1). However, the myofibrillar defects observed in response to knockdown of each complex member vary considerably in severity and reproducibility (Figure 3, Figure S1). To quantify the extent and reproducibility of defects, we took advantage of the fact that defects typically appeared as either aggregates of myosin::GFP that had a ball like appearance (Example: *pat-3*, Figure 3), as tears in myofibrils (Example: *unc-52*, Figure S1), or disruption of the normal arraying of sarcomeres (Example: *unc-89*, Figure 3). Using this classification scheme it appears that all members of the core complex (PAT-2, PAT-3, PAT-4, PAT-6, UNC-52, UNC-97 & UNC-112) are required in adult muscle to prevent the collapse of arrayed sarcomeres into ball like structures, and that the Z and M-line peripheral components (TLN-1 & ZYX-1) and M-line specific components (UNC-82 and UNC-89) are required in adult muscle to prevent disorganisation of arrayed sarcomeres. In contrast, the Z-line specific components (ATN-1 and DEB-1) do not appear to be as stringently required to prevent disorganisation or collapse of arrayed sarcomeres, inasmuch as the defects observed are not statistically significant (P>0.05, two way repeated measures ANOVA). Results from RNAi targeting the genes we selected as a potential *unc-112* interacting signalling system (*uig-1* & *cdc-42*) suggest that CDC-42 is required in adult muscle to prevent disorganisation of arrayed sarcomeres.

The variable severity we observed in disrupted sarcomere structure in adult muscle parallels the variable severity previously observed during development, where mutations in a number of the core complex genes produce both embryonic lethality and collapse of arrayed sarcomeres into ball like structures [45]. The similarity in phenotype in embryonic lethal mutants and fully developed adult muscle further supports the notion that these attachment complexes are dynamic in adult muscle. Our data also confirm past reports of the role of some of these genes in myofibril maintenance [36], [46], [47] and support the conjecture, raised in *Drosophila*, that integrin based attachment complexes, as a whole, are required for myofibrillar maintenance [48]. By using GFP fused to full-length myosin, we have observed torn myofibrils in live adult animals with acutely impaired muscle attachment complexes. These observations are consistent with previous studies of animals with mutations in two of these complex members, UNC-112 and UNC-97, where impaired resistance to mechanical damage was noted [23], [41]. Taken together, these observations appear to support the notion that the integrin attachment complexes are important in force transmission and that loss of these complexes can result in mechanical overload, and subsequent collapse, of the arrayed sarcomeres [43].

We utilized the same full length translational fusion of *gfp* to *myo-3* (myosin heavy chain A) to confirm that arrayed sarcomeres were disrupted in *unc-112^ts^* mutants (see below). We also confirmed that actin filaments are torn in fixed *unc-112^ts^* mutant animals stained with RITC-phalloidin at 24 hrs post temperature shift (not shown). In contrast to the general degradation of soluble cytosolic protein seen in *unc-112^ts^* mutant animals (Figure 1C–1F), we did not observe degradation of either myosin heavy chain or actin in western blots of *unc-112^ts^* mutant animals (not shown). Thus, the dystrophic appearance of sarcomeres in these animals largely represents dystrophy of the sarcomeres and not their degradation. These results suggest that it is degradation of soluble/freely accessible protein that is triggered by attachment complex disruption, and not degradation of insoluble/inaccessible protein. If collapsed arrays of sarcomeres can be repaired or replaced, it may be that other repair or proteolytic processes must be activated (for example in mammalian muscle, physical disruption of the sarcomere has been postulated to result in activation of alpha-crystallin mediated repair [49] and also proteasome mediated degradation [50]). Given the complexity of the sarcomere, it is quite likely that multiple proteolytic and repair processes contribute to sarcomere maintenance.

### Acute genetic disruption of muscle attachment complexes results in disorganisation and fragmentation of the mitochondrial network

**Figure 4:**
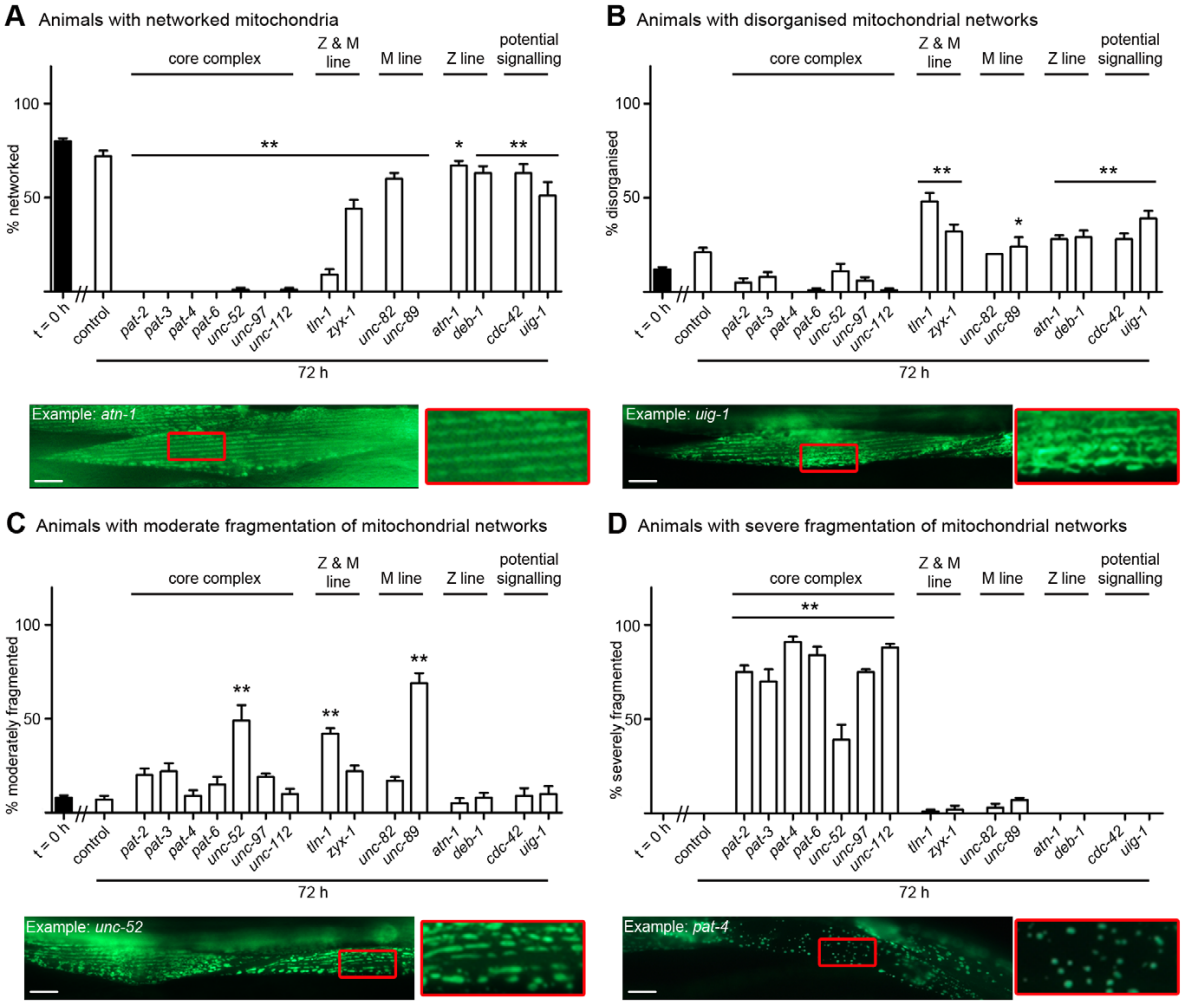
Loss of muscle attachment results in mitochondrial fragmentation. Animals expressing GFP-tagged mitochondria were age synchronised at L1 stage and grown to young adulthood at 16°C (t = 0 h). Animals were then transferred to NGM RNAi plates [87] seeded with bacteria expressing dsRNA against genes indicated for an additional 72 h (mid-adulthood) at 20°C. The 20 most Unc animals were picked and scored for identical defects in mitochondrial structure in at least two muscles within the animal and this was repeated for 5 independent RNAi treatments (n = 100 animals per condition/time point). A) Percentage of animals where only networked mitochondria were observed is displayed as average ± SEM. Below the graph is an example of an RNAi treated animal displaying networked mitochondria (as indicated by the largely continuous parallel lines of GFP), these are enlarged 300% to the right of the micrograph and look arrayed like the sarcomeres (compare to enlargement in Figure 3A). B) Percentage of animals where disorganisation of the mitochondrial network was observed is displayed as average ± SEM. Below the graph is an example of an RNAi treated animal displaying disorganisation of the mitochondrial network (as indicated by the lack of parallel lines of GFP), these are enlarged 300% to the right of the micrograph. C) Percentage of animals where moderate fragmentation (>30%) of the mitochondrial network is observed is displayed as average ± SEM. Below the graph is an example of an RNAi treated animal displaying moderate fragmentation of the mitochondrial network (as indicated by the largely non-continuous parallel lines of GFP), these are enlarged 300% to the right of the micrograph. D) Percentage of animals where severe fragmentation (>90%) of the mitochondrial network is observed is displayed as average ± SEM. Below the graph is an example of an RNAi treated animal displaying severe fragmentation of the mitochondrial network (as indicated by sparse GFP puncta), these are enlarged 300% to the right of the micrograph. A sample image of an RNAi treatment against each gene can be found in Figure S2. *, **Significant difference from control t = 72 h, (P<0.01, P<0.001 two way repeated measures ANOVA). Scale bars represent 15 µm.

Acute RNAi treatment targeting each of the muscle attachment genes also results in mitochondrial fragmentation (Figure 4, Figure S2), suggesting that the capability for energy production in muscle may be impaired [51]. For each of the RNAi treatments, the observed mitochondrial defects varied in severity and reproducibility. To quantify the extent and reproducibility of these defects, we took advantage of the fact that defects typically appeared to have different extents of fragmentation or disorganisation of the mitochondrial network. We classed animals with more than 90% loss of the mitochondrial network as severe fragmentation (Example: *pat-4*, Figure 4), those with more 30–80% loss as moderate (Example: *unc-52*, Figure 4), or as having a disorganised mitochondrial network (Example: *uig-1*, Figure 4). Using this classification scheme it appears that all members of the core complex (PAT-2, PAT-3, PAT-4, PAT-6, UNC-52, UNC-97 & UNC-112) are required in adult muscle to prevent fragmentation of the mitochondrial network (Figure 4). This is the same set of genes that is also required to prevent collapse of arrayed sarcomeres into ball like structures. Data from the other genes tested continues to show a parallel between the genes required in adult muscle to prevent sarcomere disorganisation and mitochondrial fragmentation/disorganisation with the Z and M-line peripheral components (TLN-1 & ZYX-1) and the M-line specific components (UNC-82 & UNC-89) being required in adult muscle to prevent both pathologies (Compare Figure 3A and Figure 4A). Again, RNAi against the genes we selected as a potential *unc-112* interacting signalling system (*uig-1* & *cdc-42*) produced significant defects, suggesting CDC-42 is required in adult muscle to prevent mitochondrial disorganisation. In contrast, the Z-line specific components (ATN-1 and DEB-1), which are not required for maintenance of arrayed sarcomeres, do appear to be required to prevent mitochondrial disorganisation. We also confirmed that severe mitochondrial fragmentation occurs in *unc-112^ts^* mutants (see below). Understanding how disruption of muscle attachment results in disorganisation and fragmentation of the mitochondrial network will require further studies. It could be that these defects are caused by disorganisation and/or collapse of the cytoskeleton, to which mitochondria are physically tethered [52], and/or acidification of the cytosol [53], as the result of activation of ion channels following loss of attachment to the basement membrane [54].

### Acute genetic disruption of core members of muscle attachment complexes results in collapse of arrayed attachment complexes into ball like structures

**Figure 5:**
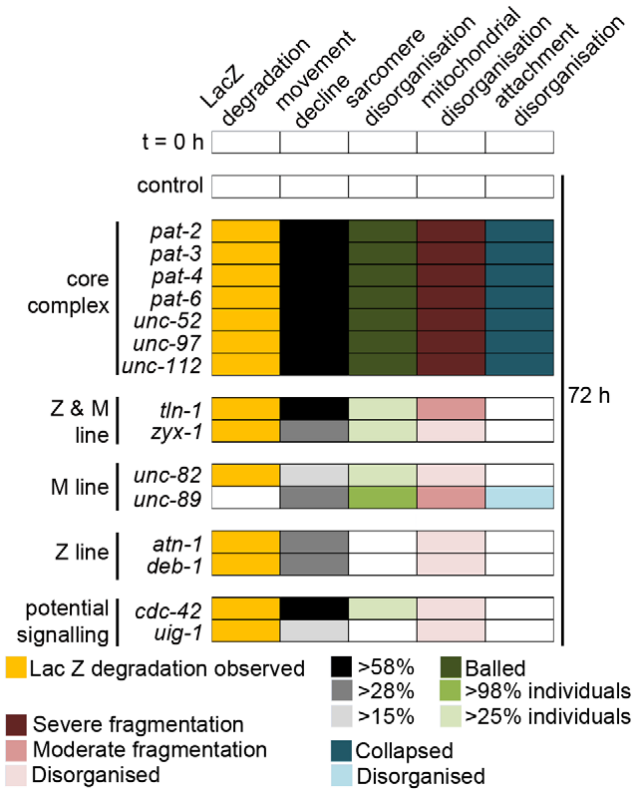
Acute loss of core integrin attachment complex members leads to collapse of attachments and multiple pathologies. Collapse of attachment complexes into ball like structures was assessed (Figure S3) and compared with the severity of other pathologies associated with attachment complex disruption (Figure 1, Figure 2, Figure 3, Figure 4, and Figures S1 and S2). Pathologies identified as significantly different from matched controls (t = 72 h, P<0.001 two way repeated measures ANOVA) were placed into identical groupings based upon severity of defect. In the case of the movement defect the extent of severity was established as follows. First, the core complex members were considered as a group and examined against the remaining components for lack of significant difference from any member of the group (t = 72 h, P>0.05 one way ANOVA). Next, the remaining components were examined for groups of components where a significant difference between individual components within a group of components did not exist (t = 72 h, P>0.05 one way ANOVA) but a significant difference between every member of the group and all other components did exist (P<0.01, one way ANOVA). Thus, the colour coding for the extent of pathology as displayed in the inset legend reflects statistically significant differences in severity of defects.

As shown in Figure 5, RNAi against the core complex members (PAT-2, PAT-3, PAT-4, PAT-6, UNC-52, UNC-97, UNC-112) results in degradation of the LacZ reporter protein, a marked decline in mobility, collapse of arrayed sarcomeres into ball like structures, and severe mitochondrial fragmentation. The severity of the sarcomere and mitochondrial phenotypes in response to knockdown of the core complex is significantly different than for non-core complex members (P<0.001, two way repeated measures ANOVA). This may suggest that more severe disruption of the attachment complexes occurs in response to these treatments. UNC-95::GFP localizes to the attachment complexes [55], allowing us to examine attachment structure, *in vivo*, in response to RNAi treatment against the attachment complex components. We find a statistically significant collapse of the arrayed attachment complexes into ball like structures in response to RNAi against the core complex members (PAT-2, PAT-3, PAT-4, PAT-6, UNC-52, UNC-97, UNC-112) but not the other peripheral components tested (Figure 5, Figure S3, P<0.001, two way repeated measures ANOVA); untreated animals display normal arraying of attachment complexes and these grow in size, but not number, with increasing age (Figure S4). Thus, it does appear that RNAi against the core complex results in a more severe disruption of the attachment complexes themselves. This disruption likely causes the collapse of the arrayed sarcomeres into similar ball like structures. Of note, RNAi against the M-line component UNC-89 also produces significant disorganisation of the attachment complexes, whereas the RNAi against the other genes tested does not (Figure 5, Figure S3, P<0.001, two way repeated measures ANOVA). It may be the case that this disorganisation accounts for the lack of LacZ degradation observed in response to RNAi against *unc-89*; further studies are clearly needed. Other relationships among the various phenotypes observed in response to attachment complex disruption are discussed below.

### Loss of DIM-1 protects against attachment complex disruption and resultant intramuscular pathologies

**Figure 6:**
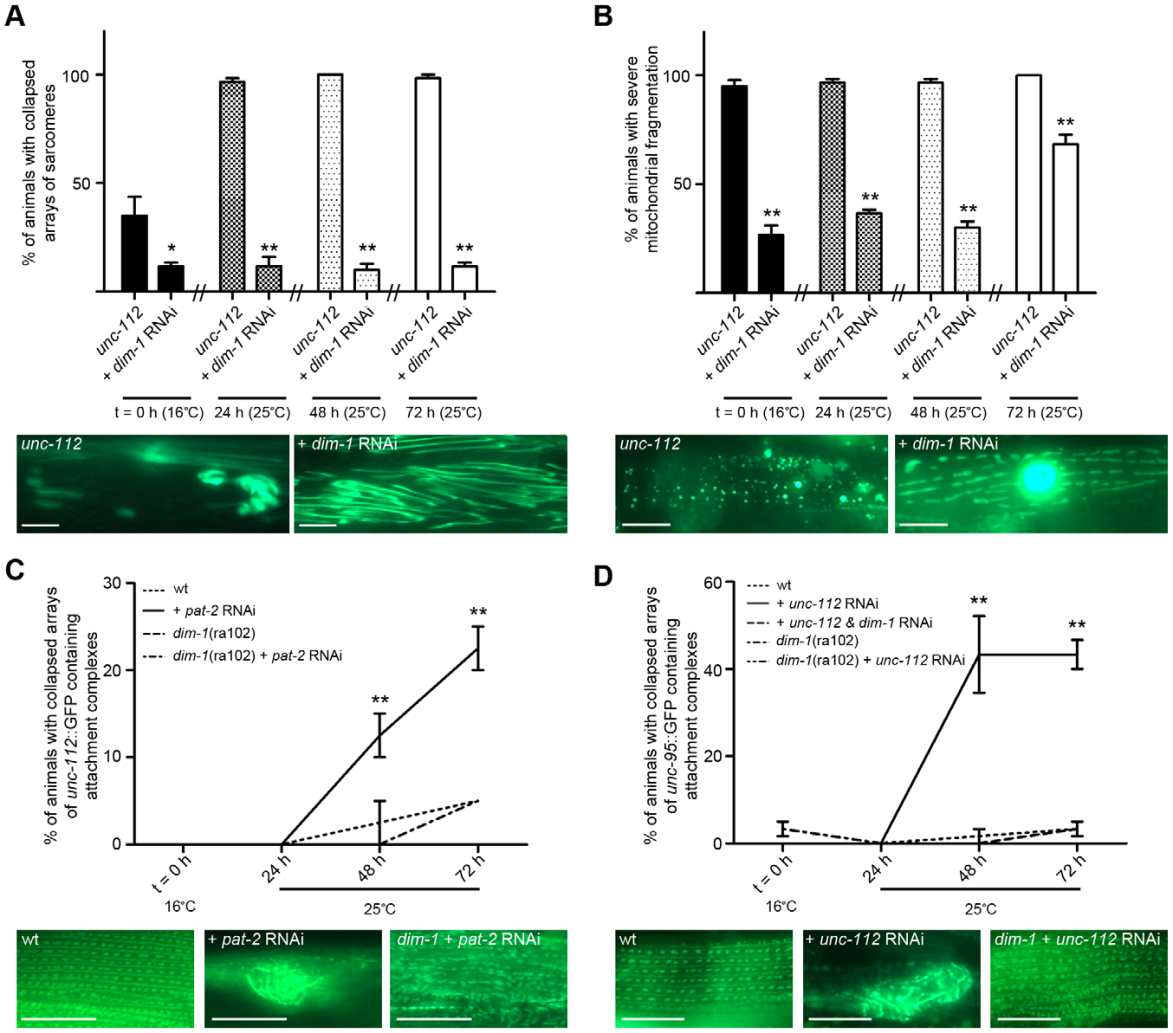
Loss of *dim-1* suppresses attachment complex collapse and resulting pathologies. Where *dim-1* RNAi was employed, indicated animals (wt or *unc-112^ts^*) were grown on RNAi targeting *dim-1* for two generations and remained on *dim-1* RNAi plates through the experiment. Age synchronised L1 stage indicated animals (wt, *unc-112^ts^*, or *dim-1(ra102)*) were gown to young adulthood at 16°C (t = 0 h) before being temperature shifted to 25°C and cultured for a further 72 h. 20 animals were picked and scored for identical pathology in at least two muscles at t = 0 h, 24 h, 48 h, and 72 h. A) Displayed are the percentage of animals containing a full length translational fusion of *gfp* to *myo-3* (myosin heavy chain A) where tearing/collapse of arrayed sarcomeres was noted (five independent experiments, n = 100). Below the graph is an example of an untreated animal displaying tearing and collapse as well as an RNAi treated animal displaying a disorganisation of arrayed of sarcomeres (for comparison see normal, disorganisation, tearing, and collapse in Figure 3 and Figure S1). *, **Significant difference from untreated (P<0.01, P<0.001) B) Displayed are the percentage of animals containing mitochondrial and nuclear localized GFP where major fragmentation of the mitochondrial network was noted (five independent experiments, n = 100). Below the graph is an example of an untreated animal displaying major fragmentation of the mitochondrial network and no visible nucleus as well as an RNAi treated animal displaying moderate fragmentation of the mitochondrial network and a GFP expressing nucleus (for comparison see networked and moderate and severe fragmentation in Figure 4 and Figure S2). **Significant difference from untreated (P<0.001). C) Displayed are the percentage of animals containing *unc-112^+^::GFP* where tearing/collapse of arrayed attachment complexes was noted (three independent experiments, n = 60). Below the graph is an example of an untreated animal displaying normal arraying of attachment complexes as well as an RNAi treated animal displaying collapse of arrayed complexes into a ball and also a *dim-1(ra102)* RNAi treated animals displaying disorganisation of arrayed attachment complexes (for comparison see normal, disorganisation, and collapse in Figure S3). **Significant difference from all other conditions (P<0.001). D) Displayed are the percentage of animals containing *unc-95::GFP* where tearing/collapse of arrayed attachment complexes was noted (five independent experiments, n = 100). Below the graph is an example of an untreated animal displaying normal arraying of attachment complexes as well as an RNAi treated animal displaying collapse of arrayed complexes into a ball and also a *dim-1* RNAi treated animals displaying disorganisation of arrayed attachment complexes (for comparison see normal, disorganisation, and collapse in Figure S3). **Significant difference between *pat-2* RNAi treated wt versus all other conditions (P<0.001). All significance values are from two way repeated measures ANOVA. Scale bars represent 15 µm.

The general correlation between the phenotypes studied, and the lack of degradation and movement decline in response to acute disruption of attachment complexes in *dim-1* mutants, prompted us to further examine if loss of DIM-1 could mitigate the effects of attachment disruption. Chronic growth of *unc-112^ts^* mutants on an RNAi feeding vector against *dim-1* was sufficient to inhibit collapse of arrayed sarcomeres into ball like structures (Figure 6A) and the extent of mitochondrial fragmentation in *unc-112^ts^* mutants (Figure 6B). In neither case were the untreated *unc-112^ts^* mutants fully normal at 16°C but in both cases the *unc-112^ts^* defects were suppressed at 16°C and following temperature upshift to 25°C. Consistent with the past report for *unc-112*; *dim-1* double mutants, we find both the arrayed sarcomeres and the attachment complexes disorganised with an appearance similar/identical to that of *dim-1* single mutants [41].

Given that either mutation in or RNAi against *dim-1* is capable of suppressing all of the effects of acute genetic disruption of attachment complexes, we next asked if *dim-1* is suppressing disruption of the attachment complexes themselves. Given that attachment complexes are disorganised (e.g. not nicely arrayed Z and M lines) in *dim-1* mutants, we opted to determine if loss of *dim-1* could prevent the collapse of attachment complexes into ball like structures (as observed in response to RNAi against the core complex members (PAT-2, PAT-3, PAT-4, PAT-6, UNC-52, UNC-97, UNC-112 (Figure 5, Figure S3)). Several lines of evidence suggest that loss of *dim-1* does prevent or delay the collapse of the arrayed attachment complexes into ball like structures. First, adults expressing UNC-112^+^::GFP display collapsed complexes following acute treatment with RNAi targeting *pat-2* (Example: +*pat-2* RNAi, Figure 6C) and the number of animals showing such disruption is significantly reduced in *dim-1* mutant animals following identical treatment in parallel (Figure 6C). Second, adults expressing UNC-95::GFP display substantial collapse of complexes following acute treatment with RNAi targeting *unc-112* (Example: +*unc-112* RNAi, Figure 6D). This is not observed in *dim-1* mutants expressing UNC-95::GFP following identical treatment in parallel (Figure 6D). Third, adult progeny of UNC-95::GFP expressing worms grown for two generations on RNAi targeting *dim-1* do not display collapsed complexes when later acutely treated with RNAi targeting *unc-112* (Figure 6D). Together, these results suggest that the disorganised array of attachment complexes observed in *dim-1* mutant animals are resistant to the effects of genetic disruption of the attachment complexes and that this decreased severity of attachment complex disruption in *dim-1* mutants accounts for the fact that *dim-1* mutants do not display protein degradation, movement decline, collapse of the arrayed sarcomeres, or severe mitochondrial fragmentation in response to RNAi treatments targeting the complexes.

### Acute genetic disruption of attachment complexes triggers calpain-mediated protein degradation

**Figure 7:**
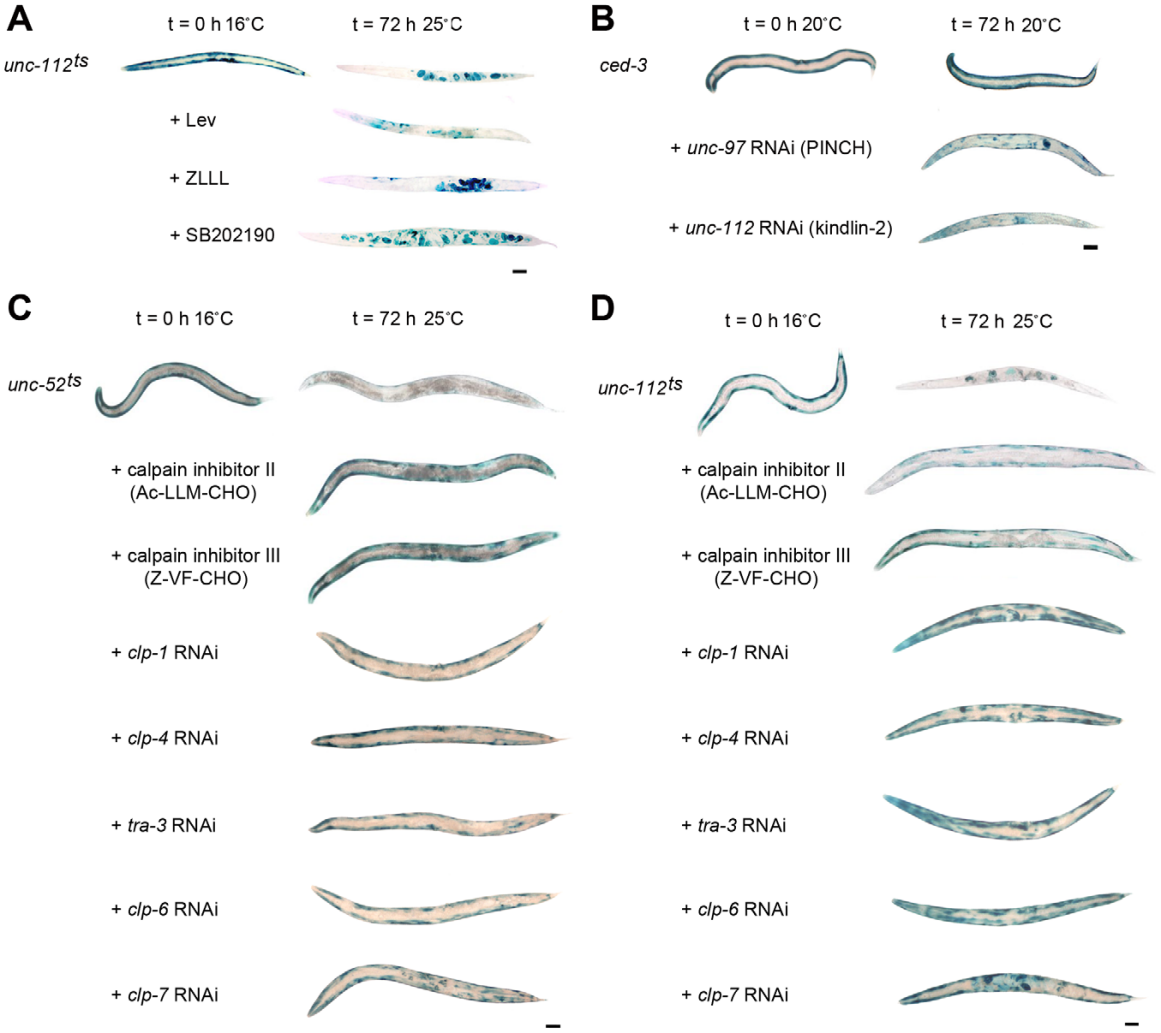
Calpain inhibition blocks protein degradation induced by acute loss of attachment. A) Age synchronised L1 stage *unc-112^ts^* mutants were gown to young adulthood at 16°C (t = 0 h) before being temperature shifted to 25°C and cultured for a further 72 h under normal conditions (top row), or on plates seeded with bacteria plus proteasome pathway inhibitors (+Lev, second row; +ZLLL, third row) or a lysosome pathway inhibitor (+SB202190, fourth row). B) *ced-3* (caspase 3) mutants were age synchronised at the L1 stage and grown to adulthood at 20°C (t = 0 h) before being cultured on RNAi plates against *unc-97* or *unc-112* for a further 72 h. In both A and B, at t = 0 h and 72 h approximately 20–30 animals were stained for β-galactosidase activity (blue). C) *unc-52^ts^* and D) *unc-112^ts^* mutants were cultured for two generations at 16°C (permissive, mutation inactive) under either normal, control conditions or on RNAi against *clp-1*, *clp-4*, *tra-3*, *clp-6* or *clp-7*. Second generation animals were then age synchronised at L1 stage and grown to young adulthood at 16°C (t = 0 h) before being temperature shifted to 25°C (non-permissive, mutation active) and cultured on the respective condition for a further 72 h. Some temperature shifted animals were also placed on calpain inhibitor II or calpain inhibitor III drug plates (5 µg/ml) at t = 0 h and cultured on drug plates for a further 72 h. Approximately 20–30 animals were stained for β-galactosidase (blue) at t = 0 h, and at 24 h, 48 h (not shown) and 72 h. All experiments were performed at least three times. The blue stain that appears as circles in the centre of t = 72 h animals is stain in muscles of developing embryos (for example *unc-112^ts^* in A and D). Scale bars represent 100 µm.

The genomic screen that identified *unc-97* and *unc-112* RNAi treatment as inducing protein degradation in adult *C. elegans* muscle demonstrated that in both cases, degradation could not be blocked by treatment with the proteasome inhibitor MG132 and occurred in mutants that block pro-autophagy signalling in *C. elegans* [29]. We confirmed and extended these results. As shown in Figure 7A, degradation in response to loss of UNC-112 was not prevented by treatment with levamisole (Lev), an acetylcholine agonist, nor by MG132 (ZLLL), a proteasome inhibitor, either of which inhibits proteasome based degradation in response to loss of motor neuron input in *C. elegans* [18]. Similarly, degradation was not prevented by SB201290, a MAPK inihibitor that blocks pro-autophagy signalling that results from an imbalance in growth factor signalling in *C. elegans* [19], [20], [37]. All compounds were used at concentrations that did block degradation in appropriate control animals and all compounds also failed to block degradation in *unc-52^ts^* mutant animals (not shown). Because autophagic degradation in response to loss of IGFR or gain of FGFR signalling also induces a severe movement defect in *C. elegans* [19], [20], [37], we tested the involvement of this pathway further. We further found that RNAi against *unc-112* or *unc-97* induces degradation in the genetic background of *mek-2* or *mpk-1* reduction of function mutations, which block signalling for autophagic degradation [19], [37], and found no increase in activated pTpY-MPK-1 MAPK in *unc-112* mutants at nonpermissive temperature (not shown). Similarly, *mpk-1* or *mek-2* RNAi did not suppress protein degradation in *unc-112^ts^* or *unc-52^ts^* mutants (these RNAi treatments did suppress degradation in *clr-1^ts^* and *let-60^ts^* mutants, not shown). Finally, N6,N6-dimethyladenosine, a direct inhibitor of autophagy [56], failed to block degradation in *unc-112^ts^* or *unc-52^ts^* mutants (this compound did suppress degradation in *daf-2^ts^* mutants, not shown). Thus, our results indicate that degradation observed in response to loss of integrin based attachment does not appear to require either the ubiquitin-proteasome or the autophagic pathways in *C. elegans*.

We therefore tested if caspases or calpains were likely to be involved. The degradation seen in response to acute RNAi treatment against *unc-97* or *unc-112* was not suppressed in a *ced-3* (caspase 3) mutant (Figure 7B). In contrast, acute treatment with calpain inhibitors did suppress protein degradation in *unc-112^ts^* and *unc-52^ts^* mutants (Figure 7C and 7D) and chronic growth on RNAi against calpain genes (*clp-1, clp-4, tra-3, clp-6* or *clp-7*) was also sufficient to block degradation in these mutants (Figure 7C and 7D). Together these results suggest that calpains are activated in response to genetic disruption of integrin containing attachment complexes and that inhibition of this activity can block the general degradation of soluble cytosolic proteins. Only *clp-1* [57] and *clp-4* [58] have previously been reported to be expressed in *C. elegans* muscle. We were therefore concerned that RNAi against one *clp* gene might result in knockdown of several *clp* gene products. However, a comparison of the nucleotide sequences of all *clp* gene primary transcripts indicates that there is no region of sequence identity that extends even to 21 nucleotides, making it unlikely that any one *clp* RNAi treatment affects multiple *clp* genes. Thus, it may be that several calpains are activated in response to genetic disruption of integrin attachment complexes in *C. elegans* muscle. Because we knew that RNAi against a combination of protease encoding genes can effectively block protein degradation in muscle [59], we tested if RNAi against a combination of *clp* genes could further suppress degradation. We failed to find additional suppression when *unc-112^ts^* mutants were treated with RNAi against *clp-1* and *clp-7*, *clp-6* and *clp-7*, or *clp-1* and *clp-6* (not shown). Our results appear to support past *in vitro* findings that calpain activity is important for the remodelling of integrin containing focal adhesion complexes [60], and speculation that calpains 1 [60], 2 [60] and 3 [61]–[64] may serve a similar function in muscle, *in vivo*. It may be that multiple calpains participate in the maintenance of integrin containing attachment complexes in metazoan muscle.

### Calpains have a role in maintenance of muscle

**Figure 8:**
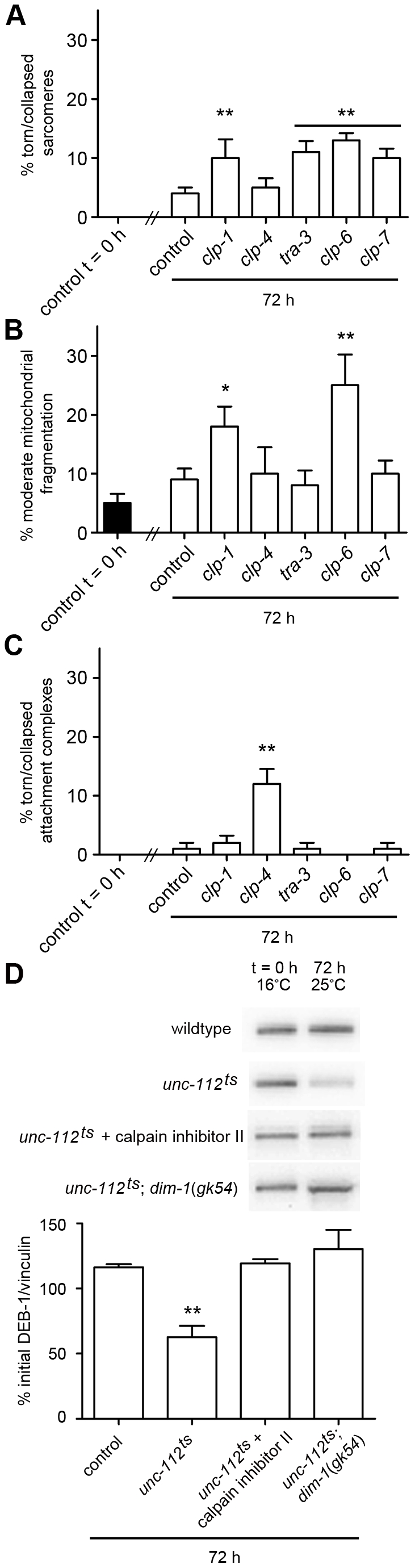
Calpains are important for maintenance of adult *C. elegans* muscle. A) Animals expressing a full length translational fusion of *gfp* to *myo-3* (myosin heavy chain A) were age synchronised at L1 stage and grown to young adulthood at 16°C (t = 0 h). Adult animals were then transferred to NGM RNAi plates [87] seeded with bacteria expressing dsRNA against genes indicated for a further 72 h to mid-adulthood. 20 random animals were picked and scored for identical defects in sarcomere structure in at least two muscles within the animal and this was repeated for 5 independent RNAi treatments (n = 100 animals per condition/time point). Displayed is the percentage of animals where torn or collapsed arrays of sarcomeres were observed (average ± SEM). Example images for each treatment can be found in Figure S5. **Significant difference from control (t = 72 h, (P<0.001)). B) Animals expressing GFP labelled mitochondria and nuclei were grown, treated and analysed as in A with the exception that mitochondrial structure was scored. Displayed is the percentage of animals where moderate fragmentation of the mitochondrial network was observed (average ± SEM). Example images for each treatment can be found in Figure S5. *, **Significant difference from control (t = 72 h, (P<0.01, P<0.001)). C) Animals expressing GFP labelled attachment complexes (UNC-95::GFP) were grown, treated and analysed as in A with the exception that attachment complex structure was scored. Displayed is the percentage of animals where torn or collapsed arrays of sarcomeres were observed were observed (average ± SEM). Example images for each treatment can be found in Figure S5. **Significant difference from control (t = 72 h, (P<0.001)). D) Wild-type, *unc-112^ts^*, and *unc-112^ts^*; *dim-1*(*gk54*) were age synchronised at L1 stage and grown to young adulthood at 16°C (t = 0 h). Adult animals were then transferred to 25°C and grown for a further 72 h to mid-adulthood. Some *unc-112^ts^* animals were also placed on calpain inhibitor II drug plates (5 µg/ml) at t = 0 h and cultured on drug plates for a further 72 h. 30 animals were picked for western blot analysis of DEB-1 levels at t = 0 h, and at 72 h. All experiments were performed at least three times. Displayed are representative western blots for each condition and a graph of the initial DEB-1 remaining at 72 h (average ± SEM for three independent experiments). **Significant difference from all other conditions (P<0.001). All significance values are from two way repeated measures ANOVA.

Given that calpains are activated in response to attachment complex disruption, we tested if calpains are important for maintenance of muscle attachment complexes. We acutely treated fully developed, wild-type adult animals with RNAi against the *clp* genes that suppressed degradation in response to attachment complex disruption and assessed the same sub-cellular structural phenotypes as previously assessed for the core complex members. As shown in Figure 8 and Figure S5, RNAi against any of the calpain genes tested resulted in defects within adult muscle. However, as was the case for all other genes tested in this work, the results were variable with respect to reproducibility and severity. Using the same classification systems as above, we find the following significant requirements for calpains in adult *C. elegans* muscle: CLP-1, TRA-3, CLP-6, and CLP-7 appear to be required to maintain arrayed sarcomeres; CLP-1, CLP-4, TRA-3, CLP-6 and CLP-7 appear to be required to prevent mitochondrial fragmentation/disorganisation (RNAi against *clp-4*, *tra-3*, and *clp-7* results in mitochondrial disorganisation, P<0.001 two way repeated measures ANOVA, not shown); and CLP-4 appears to be required to maintain arrayed attachment complexes. These observations further suggest that calpains are important to maintenance of adult muscle. As calpains appear to be needed for proper maintenance of integrin attachments and as integrin complex disruption results in activation of calpain mediated degradation, these results suggest that calpains serve a similar role in maintaining integrin attachment complexes in *C. elegans* muscle as the one they serve in maintaining focal adhesion complexes in cultured cells [60]. As *C. elegans* muscle lacks satellite cells, these results suggest a cell intrinsic role for calpains in muscle maintenance.

In cultured cells, members of the attachment complex itself are targets of calpain degradation. We therefore tested if this also appeared to be the case following calpain activation in *C. elegans* muscle. As shown in Figure 8D, DEB-1/Vinculin is degraded in *unc-112^ts^* mutants following temperature upshift and this degradation is not observed in *unc-112^ts^* mutants treated with calpain inhibitor II nor in *unc-112^ts^*; *dim-1* double mutants. It is unclear whether DEB-1 degradation is cause or effect of complex disassembly, and whether the degraded DEB-1 was in attachment complexes, in a soluble precursor pool, or both. In any case, this observation further supports the notion that calpains are activated in response to attachment complex disruption in order to facilitate attachment complex repair, and suggests that attachment complex disruption normally occurs *in vivo* in *C. elegans* muscle.

### Calpains have a role in development of muscle

**Figure 9:**
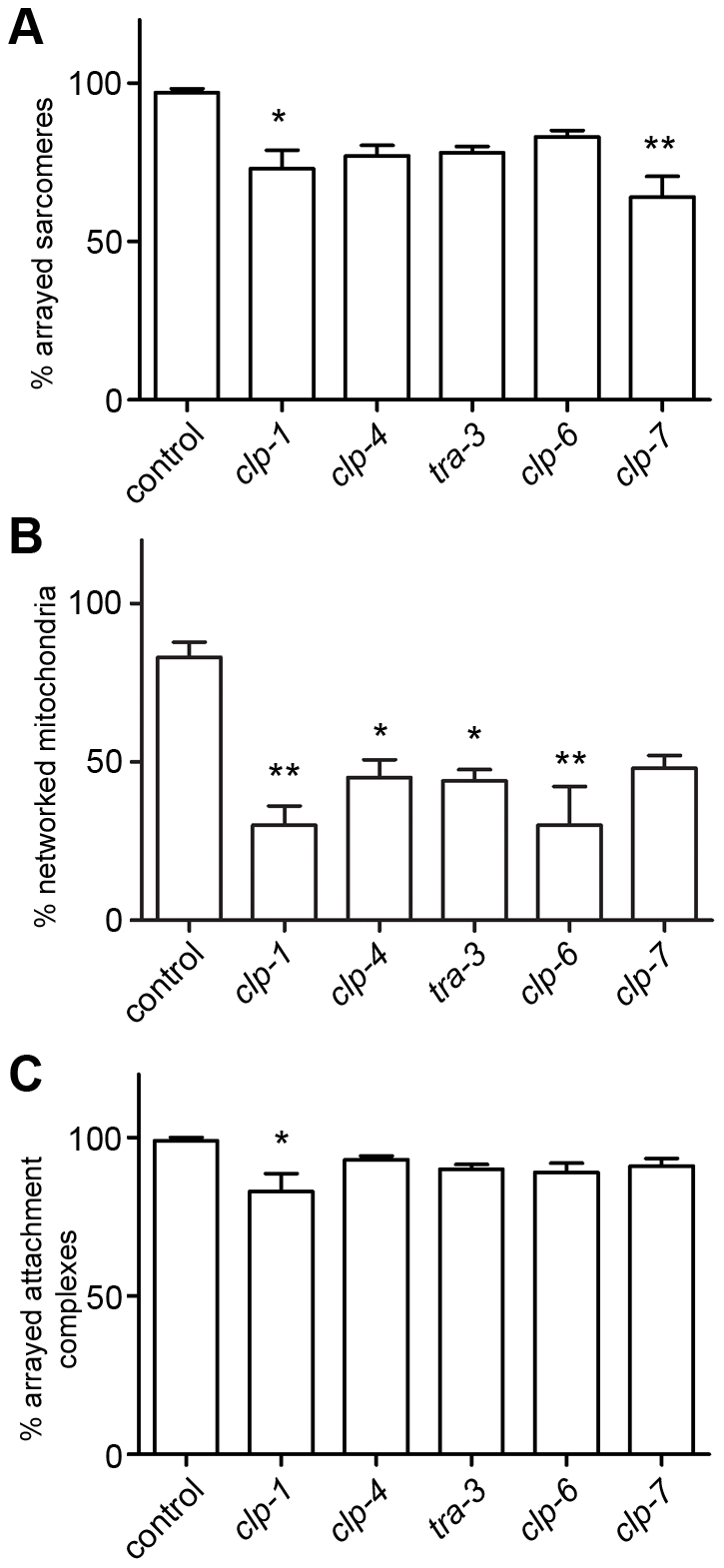
Calpains are important for development of *C. elegans* muscle. Wild-type animals expressing a full length translational fusion of *gfp* to *myo-3* (myosin heavy chain A), GFP labelled mitochondria and nuclei, or GFP labelled attachment complexes (UNC-95::GFP) were cultured from L4 stage to young adulthood under normal conditions at 20°C and on RNAi targeting *clp-1*, *clp-4*, *tra-3*, *clp-6* or *clp-7*. 20 random animals were scored for identical sub-cellular defects in at least two muscles at adulthood and 24 and 48 h post-adulthood in both the F1 and F2 generations (e.g. n = 120 per condition). A) Percentage of animals with normally arrayed sarcomeres (average ± SEM). Example images for each treatment can be found in Figure S6. *, **Significant difference from control (P<0.01, P<0.001). B) Percentage of animals with networked mitochondria (average ± SEM). Example images for each treatment can be found in Figure S6. *Significant difference from control (P<0.01). C) Percentage of animals with arrayed attachment complexes (average ± SEM). Example images for each treatment can be found in Figure S6. *Significant difference from control (P<0.01). All significance values are from one way ANOVA.

Our finding that calpains serve to maintain muscle attachment complexes and the fact that mutations in calpain 3 cause Limb Girdle Muscular Dystrophy 2A [63], prompted us to ask if the calpains also have a role in proper development of muscle. To test this, we used RNAi to knock down the identified calpain genes over two generations and examined such chronically treated adults for the same sub-muscular phenotypes as assessed in acutely treated adults. Worms developed chronically on RNAi against *clp-1, clp-4, tra-3, clp-6* or *clp-7* displayed disorganised myofibrillar, mitochondrial, and muscle attachment complex structures (Figure S6). Quantification of these defects (Figure 9) suggests a significant requirement for CLP-1 and CLP-7 for proper sarcomere development, CLP-1, CLP-4, TRA-3 and CLP-6 for proper mitochondrial development, and CLP-1 for proper integrin attachment complex formation. These results suggest that each of these calpain genes has a role in normal muscle development. Future study of animals with mutations in each of these genes may allow further dissection of specific and general requirements for calpains in *C. elegans* muscle development and physiology.

## Discussion

### Why is synthesis of integrin attachment complex proteins continuously required in terminally differentiated, adult muscle?

The integrin attachment complexes of the *C. elegans* body wall muscles serve three overlapping but partially distinct functions. First, they anchor the ends of filaments of the contractile apparatus, actin at the Z-line and myosin at the M-line, to enable proper sarcomere assembly [30], [31]. Second, they anchor body wall muscles to basement membrane just as mammalian costameres do. As hypodermal cells are also linked to the same basement membrane [30], this provides a mechanism for muscle-hypodermis communication and enables the contractile force of the muscles to be transmitted to the exoskeleton (cuticle). Third, they anchor body-wall muscles to each other. Since each longitudinal body-wall muscle band is two cells wide, both longitudinal and lateral attachments (attachment plaques) are made between muscle cells [65], [66]. These enable coordination between adjacent muscles and are thought to be akin to mammalian myotendinous junctions [67]. In metazoan muscle, it is often assumed that integrin attachment complexes must be stable in location and rigid in structure [60], [68], [69] to facilitate similar intertwined functions. However, it is not completely clear whether we should view the *C. elegans* muscle-hypodermis or muscle-muscle attachments as inert or perhaps even as stable. A worm increases about fourfold in length (250 µm to 1 mm) in growing from a first-stage larva (L1) to reproductive adulthood [70], and another 40% (to ∼1.4 mm) before growth stops. Increase in body width is approximately proportional and only 10 (of 95 total) new body-wall muscle cells are added postembryonically [71], [72]. Thus, for mobility to be maintained over this scale of growth, the muscle and hypodermal tissues must grow in a well-coordinated manner, primarily by hypertrophy rather than by proliferation. Despite the existence of careful studies of sarcomere assembly during embryonic development [30], [72], we do not yet know much about whether and how the attachment complexes undergo dynamic changes to accommodate postembryonic growth.

Here we have shown that genetic disruption of integrin containing muscle attachment complexes, by temperature-sensitive mutation or acute RNAi knockdown, results in general degradation of proteins in muscle cytosol, disruption of sarcomere organisation, fragmentation of mitochondria, and impairment of mobility. These defects occur when gene products are knocked down in fully developed, adult, muscle, so these results imply that integrin containing attachment complexes are required for maintenance of muscle. Additionally, as *C. elegans* muscle lacks satellite cells these results demonstrate that it is the attachment complexes within adult muscle cells that are required for maintenance of adult muscle. One of the most striking aspects of our findings is that these phenotypes can be induced in adults by acute RNAi knockdown. In such experiments, normal attachment complexes are present at the start of the RNAi treatment and RNAi can only lower the abundance of functionally normal proteins. We also observed that a set of phenotypes similar to those induced by adult-onset RNAi could be induced when temperature-sensitive mutants (affected in either the extramuscular ligand UNC-52 or the intramuscular attachment complex protein UNC-112) were raised to adulthood at permissive temperature, and then shifted to nonpermissive temperature. These mutants are not fully normal even when grown at nonpermissive temperature, yet the phenotypes become markedly more severe when the temperature is raised. Taken together, these observations imply that the attachment complexes must be in some measure dynamic structures. Consistent with this, a recent study has shown that some proteins associated with *C. elegans* sarcomeres do display dynamic exchange *in vivo* [73]. Our examination of animals labelled with an UNC-95::GFP fusion, which does display dynamic exchange [73], suggests that as the adult animals grow, the number of Z-line attachment complexes remains constant or increases only slightly, while the spacing between adjacent complexes increases to accommodate body growth (e.g. the Z-line to M-line distance is the same but the Z-line to adjacent Z-line distance is increased). This observation is in line with past observations that the number of sarcomeres remains constant after the last larval stage [30]. Both observations are most consistent with a model in which each Z-line attachment complex as an entity is maintained over time, but undergoes continuous dynamic exchange and/or accretion of some constituent proteins to accommodate animal growth. In the case of UNC-95, the data suggest that it undergoes dynamic exchange [73] and also accretion (as evidenced by the growing area of UNC-95::GFP labelled Z-line complexes (Figure S4)). Thus, strong effects on phenotype might be produced if RNAi depleted the free pool of such an attachment complex protein so as to compromise either exchange or accretion. Additionally, one might predict that the increased distance between adjacent Z-line attachment complexes would alter the tension on each Z-line attachment complex; this has previously been suggested [74], [75] and is postulated to increase roughly linearly with postembryonic growth [43]. Continuing protein accretion may make the attachment complexes more robust to withstand this additional tension.

### How is loss of DIM-1 suppressing collapse of arrayed attachment complexes?

The mechanism of mechanical coupling between adjacent Z-lines in *C. elegans* is currently unknown. DIM-1 is localized around the Z-line attachment complexes, so it is possible that DIM-1 is required to allow for mechanical coupling between adjacent Z-lines. The fact that normal arrays of Z and M-lines and of sarcomeres are not typically seen in the absence of DIM-1 suggests decreased mechanical coupling must occur. Loss of *dim-1* suppresses the protein degradation, collapse of arrayed sarcomeres and attachment complexes, fragmentation of mitochondria, and movement defects we observed in response to integrin attachment complex disruption. The suppression of these phenotypes by mutation in or RNAi targeting *dim-1* suggests, but does not prove, that it is additional mechanical strain placed upon the integrin attachment complexes that causes them to fail in response to acute gene knockdown of protein complex members. Failure of attachment complexes through a combination of decreased gene function and mechanical strain has been observed in animals with embryonic lethal mutations in members of this complex [45]. In these worms, mechanical strain in the form of onset of muscular contraction is known to be the cause of failure of integrin attachment complexes. Though the precise molecular composition and functions of these complexes are different in embryos and adults, the consequences of failure are strikingly similar, inasmuch as the micrographs of myosin in these embryonic mutants look very much like the MYO-3::GFP “balling” phenotype we observed in response to some acute RNAi knockdowns. Clearly, further work on *dim-1* is required to fully understand how *dim-1* mutants suppress the pathologies observed in response to genetic disruption of integrin attachment complexes in adult muscle.

### Why are calpains activated in response to integrin complex disruption?

Whereas mutations in or RNAi against *dim-1* suppress all of the pathologies observed in response to genetic disruption of the integrin attachment complexes, RNAi against genes encoding calpains, or treatment with calpain inhibitors, suppresses the degradation of cytosolic proteins but not the movement defect. By contrast proteasome inhibitors, autophagy inhibitors, or a caspase mutation fail to block the degradation. These data suggest that calpains are activated in response to integrin attachment complex disruption. The data show that calpains are necessary for the degradation observed in response to genetic disruption of integrin attachment complexes in fully developed muscle, but do not show if calpain activation is sufficient.

Calpains are normally ascribed a role in partial rather than complete degradation of proteins [76]. For example, partial degradation of integrin attachment complex member proteins by calpains is believed to have a role in both assembly and disassembly of these complexes [60]. The calpain activation we infer could be solely responsible for the degradation of cytosolic protein as the result of catastrophic failure of the attachment complexes (e.g. inability to reassemble/repair the complexes); this is the simplest explanation. However, it is also possible that the calpain activation could be required to signal increased degradation via another or a combination of other proteolytic systems. For example, calpains (CLP-1 and TRA-3) and lysosomal proteases (ASP-3 and ASP-4) are required sequentially in *C. elegans* neurons undergoing excitotoxic cell death [57]. In the present study, RNAi against single calpain genes *clp-1*, *clp-4*, *tra-3*, *clp-6*, or *clp-7* is sufficient to suppress degradation. Thus, any potential sequential activation of proteases in response to genetic disruption of integrin attachment complexes is at least partially distinct from the sequential activation observed in excitotoxic cell death in *C. elegans* neurons. Whether or not calpain activation is solely responsible for general degradation of cytosolic protein in response to genetic disruption of integrin attachment complexes, the fact that calpain activation is known to promote remodelling of such attachment complexes [60] suggests that this is the reason for initial activation of the calpains. Consistent with calpains serving to maintain integrin attachment complexes in adult muscle, we found that treating fully developed adult muscle with RNAi against the calpains results in attachment complex disruption, defects in the normal arraying of sarcomeres, and mitochondrial fragmentation. Additionally, we found that calpains do degrade the attachment complex member DEB-1/Vinculin when the complex is disrupted. Thus, in adult *C. elegans* muscles, integrin attachment complexes and calpains appear to participate in a feedback system whereby integrin attachment complex disruption can activate calpains and calpain activation facilitates repair/remodelling of disrupted complexes. This system allows these complexes to carry out their intertwined functions, including coordinated growth. In support of this suggestion, one of the calpain genes that participates in this system, *tra-3*, was recently shown to be mutated in a natural variant strain that fails to alter growth in response to temperature [77]. More work is required to understand the specific roles these calpains serve *in vivo* and also the interrelationship(s) between them.

### What is causing the decline in movement in response to attachment complex disruption?

While it is tempting to suggest that the cytosolic degradation, sarcomere disorganisation, or mitochondrial fragmentation is causing the movement decline we observe, each individual defect has previously been shown to be sufficient to cause a movement decline [20], [78]–[80]. Thus, which particular subcellular damage(s) leads to the organismal level mobility defect may be somewhat specific to each RNAi treatment, and each may in fact be the result of quantitatively different effects in multiple subcellular compartments within different individuals receiving the same RNAi treatment. For example, given the extent of intra-muscular pathology observed in response to RNAi against a core complex component, a very severe movement decline is entirely expected if each defect is sufficient to cause a movement decline and each effect is additive. Similarly, it is not particularly surprising that when both the sarcomeres and mitochondria show defects, the movement decline is significantly greater in animals with more severe mitochondrial disruption (for example *tln-1*). Conversely, it may be somewhat surprising that RNAi against *cdc-42* produces a severe movement decline, as it does not produce similar severities of sub-cellular pathologies (as the core complex and *tln-1*) and also quite surprising that RNAi against *unc-89* produces only a modest decline in movement despite producing highly disorganised arrays of sarcomeres and attachment complexes and substantial fragmentation of mitochondria. Thus, while a clear relationship exists for the core complex as a group, the more peripheral components of the complex appear to have more individual differences, perhaps as the result of more specialized functions of each gene product. Future prospective studies of individual animals where defects in multiple sub-muscular compartments are followed in parallel with movement decline may shed further light on this question, as may further studies of genes for which RNAi treatments produce unique patterns of sub-cellular defects (for example *unc-89*). Additionally, as different components of these attachment complexes have been shown to have different kinetics of exchange within these complexes [73], it could prove quite interesting to conduct studies which attempt to correlate the relative severity of movement defect and/or subcellular pathology with the kinetics of exchange. Regardless of the complexity, it seems clear that loss of integrin attachment complexes in adult muscle has multiple subcellular consequences, which usually result in decline in movement that is related to the extent of intramuscular damage.

### How is the general degradation seen in response to integrin complex disruption relevant to human health and/or disease?

We started these studies because two members of an integrin attachment complex showed decreased mRNA expression in response to spaceflight [22], a condition associated with disuse atrophy, and because RNAi targeting these genes provoked general protein degradation within muscle [29]. Here we have shown that acute genetic disruption of the core members of muscle integrin attachment complexes results in activation of calpains, and we have argued that the main physiologic role of this activation is to facilitate repair and/or replacement of damaged complexes. It should prove a matter of much interest to determine if attachment complex disruption occurs in higher animals and if calpains serve a similar muscle intrinsic repair/remodelling role. It may be that the Z-line streaming observed in human muscle subjected to prolonged physical forces [81] is a hint that these complexes can be damaged and repaired in higher animals. If calpains serve such a role in higher animals, then it is almost certainly the case that the general degradation seen in response to integrin complex disruption is transient and thus the main role of this degradation is in facilitating repair/remodelling, not in causing muscle atrophy. However, it is likely that in conditions of prolonged disruption of these attachment complexes, calpain activation and the consequent general degradation would be sustained. It may be the case that the general degradation we observe is relevant to muscle wasting seen in the congenital myopathy in individuals with a mutation in an integrin receptor gene [82]. Additionally, our results add support to the notion that lack of calpain activity accounts for part of the progressive dystrophy noted in rodent calpain mutants [62] and in humans suffering calpain mutations [63].

### Moving toward an integrated picture of the control of muscle protein degradation *in vivo*

Here we have reported that genetic disruption of integrin based attachment to the basement membrane induces calpain activation and subsequent general degradation of cytosolic protein content. This is the fourth cell surface receptor on *C. elegans* muscle identified as a regulator of proteolysis and the third intramuscular proteolytic system shown to be regulated by extramuscular signals [18]–[20]. Thus, we are moving closer to an integrated picture of how *C. elegans* muscle co-ordinately maintains cytosolic protein content in response to external cues. All signals identified to date have human orthologs [83], suggesting that some knowledge of integrated control of human muscle protein content can be gleaned from *C. elegans*.

## Materials and Methods

### Nematode growth, genetics, and transgenics

Nematode strains were maintained and grown using standard *C. elegans* culturing techniques [84] at 20°C or, in the case of temperature sensitive mutants at 16°C, using the *Escherichia coli* strain OP50 as food source. Genetic constructions were conducted using standard techniques. Mutant alleles used in this work were as follows: LG I: *mek-2(ku114)*; LG II: *unc-52(e669su250^ts^)*; *clr-1(e1475^ts^)*; LG III: *daf-2*(*m41^ts^*); *mpk-1(n2521)*; LG IV: *ced-3(n717)*; *cha-1(p1182^ts^)*; *let-60(ga89^ts^)*; LG V: *unc-112(r367^ts^)*; and LG X: *dim-1* (*gk54*) and (*ra102*). The transgenes used in these experiments were as follows: *ccIs55* (*sup-7*(*st5*); *unc-54::lacZ;* integrated on LG V) for assessing protein degradation with histology as previously described [17]; *jIs01* (*rol-6*(*su1006*); *myo-3::GFP*; unknown site of integration) for visualising sarcomeres with fluorescent microscopy [38], *ccIs4251* (pSAK4 (myo-3 promoter driving mitochondrially targeted GFP); pSAK2 (myo-3 promoter driving a nuclear-targeted GFP::LacZ fusion); and a *dpy-20* subclone; integrated on LG I) [85] and *zcIs14* (*myo-3::GFP^mt^*; unknown site of integration) [86] for visualising the mitochondria with fluorescent microscopy; *ryIs22* (*rol-6*(*su1006*); *unc-95::GFP*; integrated on LG X) [55] and *raEx16* (*rol-6*(*su1006*); *unc-112^+^::GFP*) [24] for visualising the muscle attachment complexes with fluorescent microscopy, these encode GFP tagged UNC-95 and UNC-112 respectively; and *dvIs511* (pCL197(*Pmyo-3::Ub-G76V-GFP*); pCL148(*Pmyo-3::DsRed monomer*); integrated on LG I) (gift from Chris Link, University of Colorado at Boulder), and *njEX38* (*pG_o_::GFP*, *rol-6*(*su1006*), *punc-54::daf-2^+^*) [20] for visualising any loss of cytosolic protein due to membrane disruption, these include DsRed and GFP constructs expressed in the body wall muscle cytosol. *raEx16* was also used in the *unc-112^ts^* rescue experiments.

### Development of animals on RNAi

Culturing was essentially as described [87]. For chronic treatment with RNAi four L4 larvae were transferred to standard RNAi plates with bacterial lawns expressing double-stranded RNA for the relevant genes. Most bacterial lawns expressing double-stranded RNA were grown from bacterial clones from the MRC Ahringer Library [87]. Bioinformatic work was conducted utilizing WormBase [88]. MRC clones used were as follows: *atn-1*: V-8I08, *cdc-42*: II-5P13, *Y71G12B.11*: I-8K21, *pat-2*: III-4P15; *pat-4*: III-1C19; *pat-6*: IV-1E21; *uig-1*: V-8D12; *unc-52*: II-9A20; *unc-89*: I-1B22; *unc-97*: X-3I11; *unc-112*: V-9L03; *zyx-1*: II-8H13; *clp-1*: III-4O15; *tra-3*: IV-7D13; *clp-6*: IV-1D01; *clp-7*: IV-1B23; *mek-2*: I-7L20; *mpk-1*: III-2I01. Bacterial lawns expressing double-stranded RNA targeting *clp-4* or *deb-1* were, respectively, grown from a bacterial clone which was kindly provided by Chris Link (University of Colorado at Boulder) or from a clone from the Open Biosystems Vidal Library [89], clone 10002-B11. Bacterial lawns expressing double-stranded RNA targeting *pat-3* were grown from a bacterial clone produced for this work. Briefly, an L4440 plasmid containing an ∼1.8 kB cDNA fragment of *pat-3* (BamHI digestion product from a Yuji Kohara cDNA clone, provided by Hiroshi Qadota (Emory University)) was transfected into HT115 (ΔlacZ, produced by mutagenesis) and confirmed as described [87]. Behavioural, developmental, and sub-cellular phenotypes were scored in the F_1_ and F_2_ generations as described [29]. Sub-cellular phenotypes were scored as described above at early adulthood and 24, 48, and 72 hours later. In experiments where RNAi against a gene was tested for ability to suppress degradation or subcellular pathology (e.g. *mpk-1*, *mek-2*, *clps*, and *dim-1* in *unc-112^ts^* or *unc-52^ts^* mutants or in wt), strains were grown on RNAi for at least two generations prior to the onset of acute temperature shift experiments (e.g. experiments were always conducted in F_3_ or later generations).

### Acute treatment of adult animals with RNAi

Animals were roughly age-synchronised as described [17]. Animals were then re-plated to fresh OP50 bacterial lawns and grown at 16°C for 48–60 hours to early adulthood. Subsequently, animals were manually transferred to standard RNAi plates seeded with standard *E. coli* HT115 RNAi feeding vector(s) [87], as described above. Transgenic animals were analysed for protein degradation, sarcomere defects, mitochondrial defects, or attachment complex abnormalities at 24, 48 and 72 hours post-adulthood.

### Microscopy

All images were captured on either a Leitz Orthoplan with Leica DFC300F digital camera and Leica Firecam software (Pittsburgh), a Nikon H600L with a Nikon Digital Sight DS-Fi1 digital camera and proprietary software (Nottingham), or a Zeiss AX10 with an Axiocam MRC digital camera and Axiovision LE software (Nottingham). Confocal images (Pittsburgh) were obtained on Leica TCS-SP5 or Olympus FV1000 microscopes and analysed with ImageJ software. Other image analysis and figure preparation was conducted in Photoshop. RITC-phalloidin was used as previously described [37].

### Acute activation of temperature-sensitive mutant animals

Animals were roughly age-synchronised as described [17]. Animals were then re-plated to fresh OP50 bacterial lawns or fresh RNAi clone lawns and grown at 16°C for 48–60 h to early adulthood. Subsequently, animals were transferred to 25°C; in the case of drug experiments animals were placed on either OP50 or drug plates immediately prior to temperature upshift. Transgenic animals were analysed for protein degradation, sarcomere defects, mitochondrial defects, or movement defects at 24, 48 and 72 h post-adulthood.

### Immunoblotting

Western blots in Pittsburgh were conducted as previously described [19], [20], [37], [38] utilizing the following primary antibodies: anti-β-galactosidase JIE7, anti-myosin heavy-chain A 5–14, and anti-actin JLA20 [90], all from Developmental Studies Hybridoma Bank, University of Iowa, USA; and anti-pTpY-ERK 9101S from Cell Signalling Technologies. Peroxidase-conjugated donkey anti-mouse and donkey anti-rabbit secondary antibodies were from Jackson Immunoresearch. For analysis of myosin and actin degradation in *unc-112^ts^* mutants, blots were probed for both β-galactosidase and actin or myosin so that a direct comparison of degradation could be made.

Western blots in Nottingham were conducted as follows: 30 worms were picked into 13 µl M9 Buffer and 7 µl 3×Laemmli buffer was added prior to boiling for 5 min. Lysates were then frozen at −20°C until analysis. Subsequently, samples were thawed on ice, vortexed thoroughly and loaded onto precast 18-well 12% sodium dodecyl sulfate polyacrylamide electrophoresis gels (Criterion XT Bis-Tris; Bio-Rad, Hemel Hempstead, UK) and run at 200 V for 1 h. After equilibration in transfer buffer for 15 min, the gel was transferred on ice at 100 V for 45 min to a methanol pre-wetted 0.2 µm Immobilon PVDF membrane (Millipore). Next, the membrane was blocked in 5% (w/v) BSA in TBS-T (Tris Buffered Saline and 0.1% Tween-20) for 1 h at room temperature and then incubated overnight at 4°C in primary β-galactosidase antibody (Promega) or DEB-1 antibody ([91], Developmental Studies Hybridoma Bank) at a 1∶40,000 dilution or 1∶1,000 (respectively) in 5% (w/v) BSA in TBS-T. The following morning the membrane was washed (3×5 min) in TBS-T and then incubated in peroxidase-conjugated donkey anti-mouse secondary antibody (R&D systems) at a 1∶16,000 dilution in 5% BSA/TBS-T for 1 hour at room temperature. After further washes (3×5 min) in TBS-T the membrane was developed using Immunstar ECL reagent (Bio-Rad) for 5 min and the protein bands visualised on a Chemidoc XRS system (Bio-Rad). Peak densities of the bands were statistically analysed by two-way repeated measures ANOVA using GraphPad Prism 5.

### Use and confirmation of *in vivo* activity of pharmacologic agents

Cycloheximide (CHx) was used as previously described on 5 ml plates [17]. Confirmation that the cycloheximide was active was achieved by confirming the lack of larval development in the F_1_ generation. Levamisole (Lev) and MG132 (ZLLL) were used as previously described on 5 ml plates [17], [18]. Confirmation that levamisole and MG132 were active was achieved by noting block of degradation in *cha-1^ts^* animals at 34 hours post temperature upshift. SB201290 was used as previously described on 5 ml plates [20]. Confirmation that SB201290 was active was achieved by noting block of degradation in *clr-1^ts^* or *let-60^ts^* animals at 48 or 72 hours post temperature upshift, respectively. N6, N6-dimethyladenosine (Toronto Research Chemicals) was used at 0.5 mM. Confirmation that N6, N6-dimethyladenosine was active was achieved by noting block of degradation in *daf-2^ts^* mutants at 48–72 h after a shift to 25°C. Calpain inhibitors II and III (BioChemica) were prepared as 5 mg/ml stock solutions in DMSO. These stock solutions were added to the bacterial lawn of seeded NGM plates as 1∶1000 dilutions (typically 5 µl on a 5 ml plates) and allowed to dry 1–3 days prior to use. Animals were picked directly onto the location of seeding with the drug. DMSO only vehicle control plates were prepared similarly.

### Movement analysis

Movement analysis was conducted essentially as described [19], [20]. All experiments were conducted by a single individual to prevent individual to individual differences in scores. For Figure 2A (BF, Pittsburgh): Wild-type or *unc-112^ts^* animals were temperature shifted as indicated and movement assessed at the indicated times. (*unc-112^ts^/+*heterozygotes were generated by crossing homozygous *unc-112^ts^* hermaphrodites with *mIs10* (P*myo-2::GFP;* integrated on LG V) males and collecting F1 hermaphrodites with pharyngeal GFP). Five animals were picked and assessed 10 times for a total of 50 independent measurements for each genotype at each timepoint. For Figure 2B and 2C (TE, Nottingham): Wild-type (*ccIs55*) or *dim-1(ra102)* animals were treated acutely with RNAi and rates of movement were assessed at young adulthood (t = 0 hour) and mid-adulthood (72 hours post RNAi treatment) time points. Animals were individually picked into 10 µl M9 buffer. The number of sinusoidal movement patterns completed over 10 seconds was recorded and multiplied to obtain movement rate per minute. This was repeated 10 times for each animal to give a total of 100 measures per experimental treatment. In the case of severe movement defects where there was an absence of sinusoidal movements due to paralysis, the number of times the head of the animal moved from left-to-right and left again was measured. Movement data were analysed for each strain by two-way repeated measures ANOVA in GraphPad Prisim5.

## Supporting Information

Figure S1 Acute loss of muscle attachment causes disorganisation and collapse of arrayed sarcomeres. Animals expressing a full length translational fusion of *gfp* to *myo-3* (myosin heavy chain A) were age synchronised at L1 stage and grown to young adulthood at 16°C (t = 0 h). Adult animals were then transferred to NGM RNAi plates [87] seeded with bacteria expressing dsRNA against genes indicated for a further 72 h to mid-adulthood. Displayed are sample images of defects in sarcomere structure observed for each indicated treatment. White arrow, minor sarcomere disorganisation (Examples: *atn-1*, *deb-1*); yellow arrow, major sarcomere disorganisation (Examples: *unc-82*, *tln-1*); red arrow, balled array of sarcomeres (Example: *unc-112*), red asterisk, torn array of sarcomeres (Example: *unc-52*). Scale bar represents 50 µm. Quantification of defects can be found in Figure 3; note that minor disorganisation (e.g. white arrows) was classed as normal for the purposes of quantification, whereas major disorganisation (e.g. yellow arrows) was classed as disorganised.
(TIF)

Figure S2 Acute loss of muscle attachment results in mitochondrial fragmentation. Animals expressing GFP-tagged mitochondria and nuclei were age synchronised at L1 stage and grown to young adulthood at 16°C (t = 0 h). Animals were then transferred to NGM RNAi plates [87] seeded with bacteria expressing dsRNA against genes indicated for an additional 72 h (mid-adulthood) at 20°C. Examples of networked, disorganised, and moderately and majorly fragmented mitochondria are displayed in Figure 4, where quantification of these defects can also be found. Minor fragmentation is usually classed as disorganised (for example the t = 72 h control image shows minor fragmentation on either side of the nucleus). Scale bar represents 50 µm.
(TIF)

Figure S3 Acute genetic disruption of attachment complex genes results in disorganisation and collapse of attachment complexes. Animals expressing GFP-tagged attachment complexes (UNC-95::GFP) were age synchronised at L1 stage and grown to young adulthood at 16°C (t = 0 h). Animals were then transferred to NGM RNAi plates [87] seeded with bacteria expressing dsRNA against genes indicated for an additional 72 h (mid-adulthood) at 20°C. The 20 most Unc animals were picked and scored for identical defects in attachment complex structure in at least two muscles within the animal and this was repeated for 5 independent RNAi treatments (n = 100 animals per condition/time point). A) Percentage of animals where only normal arrays of attachment complexes were observed is displayed as average ± SEM. Below the graph is an example of an RNAi treated animal displaying normal arrays of attachment complexes (as indicated by straight parallel lines of GFP), these are enlarged 300% to the right of the micrograph. Note that Z-line attachment complexes, termed dense bodies, appear as punctate lines (white arrow) while M-line attachment complexes appear as a continuous line (yellow arrow). B) Percentage of animals where disorganisation of arrayed attachment complexes were observed is displayed as average ± SEM. Below the graph is an example of an RNAi treated animal displaying disorganised attachment complex arrays (as indicated by lack of straight parallel lines of GFP), these are enlarged 300% to the right of the micrograph. C) Percentage of animals where sarcomere arrays have collapsed into ball like structures is displayed as average ± SEM. Below the graph is an example of an RNAi treated animal displaying a collapsed array of sarcomeres (as indicated by GFP that appear as circular lines rather than straight lines, see also Figure 6C and 6D for examples), these are enlarged 300% to the right of the micrograph. **Significant difference from control t = 72 h, P<0.001 (two way repeated measures ANOVA). Scale bars represent 15 µm.
(TIF)

Figure S4 Z-line attachment complexes increase in area as *C. elegans* grow larger post-adulthood. Live worms of a strain carrying an *unc-95*::*gfp* fusion were examined by confocal microscopy at the fourth larval stage (t = −24 h, worm length = 0.75±0.02 mm), one day later as young adults (t = 0 h, length = 1.03±0.03 mm) or an additional two days later (t = 48 h, length = 1.44±0.02 mm). Images (7–9 worms of each group) were taken of a muscle cell in an approximately constant position (just posterior to the vulva, inner or outer cell in a band), fragments of adjacent cells and the M-lines removed manually in Photoshop, and the fluorescent Z-line attachment complexes (dense bodies) counted and their sizes measured using ImageJ software. The number of dense bodies per cell was t = −24 h, 333±26; t = 0 h, 412±24; t = 48 h, 402±31; we cannot state with high confidence whether or not the number of dense bodies per cell changes. The arrows indicate the medians of the dense body area distributions for t = −24 h (median area = 0.23 µm^2^, n = 3002), t = 0 h, young adults (median = 0.35 µm^2^, n = 2888) or t = 48 h (median = 0.71 µm^2^, n = 2813). In some rare instances, Image J fails to separate adjacent bodies, leading to an artifactually high size measurement (>1.5 µm^2^, at right); this distorts means but has little effect on medians. All pairwise comparisons show that differences between these size distributions are highly significant (P<0.0001, 2-tailed T-tests with unequal variances).
(TIF)

Figure S5 Calpains are important for maintenance of adult *C. elegans* muscle. A) Animals expressing a full length translational fusion of *gfp* to *myo-3* (myosin heavy chain A) were age synchronised at L1 stage and grown to young adulthood at 16°C (t = 0 h). Adult animals were then transferred to NGM RNAi plates [87] seeded with bacteria expressing dsRNA against genes indicated for a further 72 h to mid-adulthood. B) Animals expressing GFP labelled mitochondria and nuclei were grown and treated as in A. C) Animals expressing GFP labelled attachment complexes (UNC-95::GFP) were grown, treated and analysed as in A. Quantification of defects can be found in Figure 8. Scale bars represent 15 µm.
(TIF)

Figure S6 Calpains are important for development of *C. elegans* muscle. A) Animals expressing a full length translational fusion of *gfp* to *myo-3* (myosin heavy chain A) were cultured from L4 stage to young adulthood under normal conditions at 20°C and on RNAi targeting *clp-1*, *clp-4*, *tra-3*, *clp-6* or *clp-7*. B) Animals expressing GFP labelled mitochondria and nuclei were grown and treated as in A. C) Animals expressing GFP labelled attachment complexes (UNC-95::GFP) were grown, treated and analysed as in A. Quantification of defects can be found in Figure 9. Scale bars represent 15 µm.
(TIF)
