# Supplementary figures and images for: Calpains Mediate Integrin Attachment Complex Maintenance of Adult Muscle in Caenorhabditis elegans
Source: PLoS Genet. 2012 Jan 12;8(1):e1002471. doi: 10.1371/journal.pgen.1002471 (PMC3257289; doi:10.1371/journal.pgen.1002471)

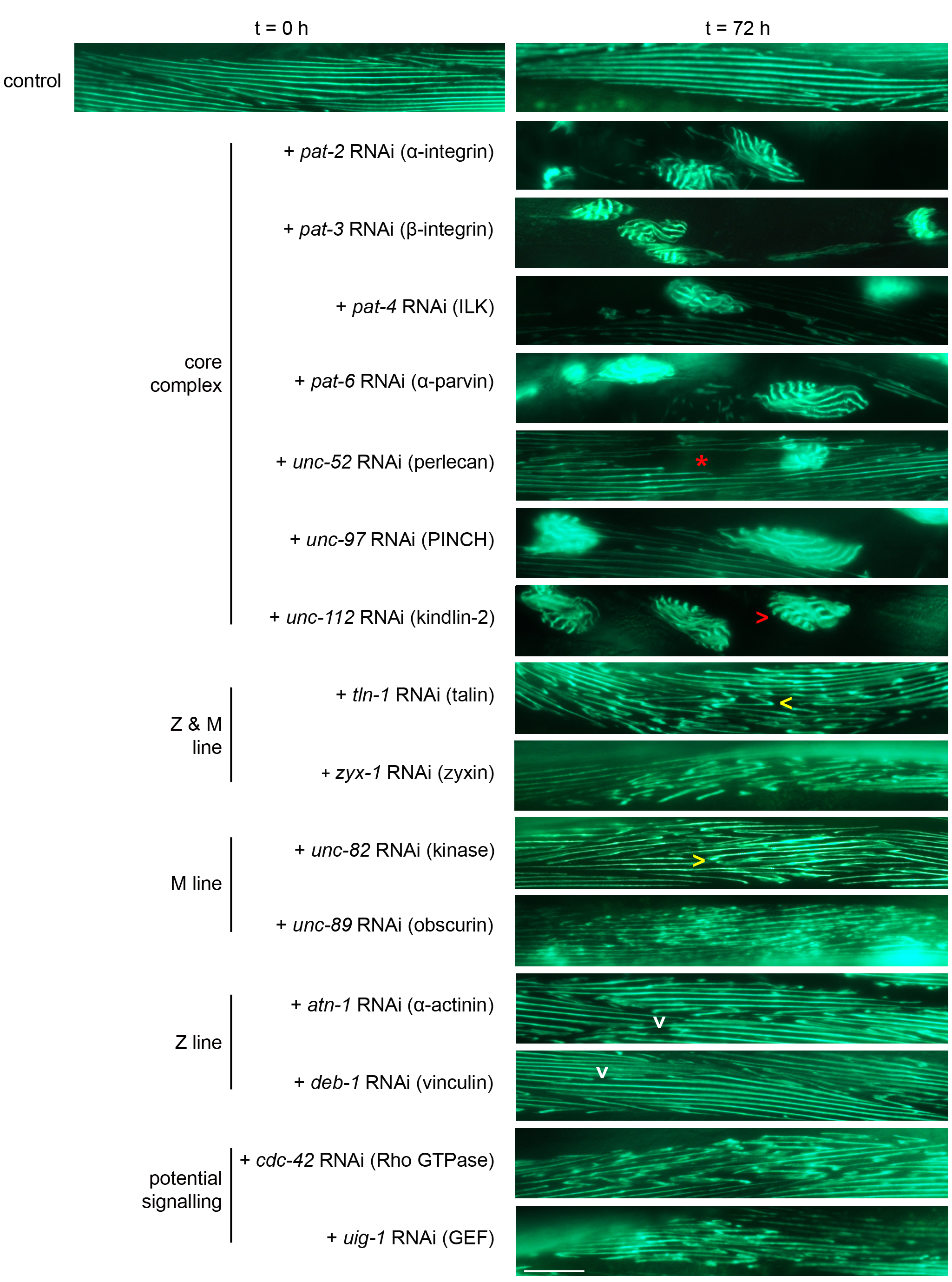

Supplement: Figure S1 — Acute loss of muscle attachment causes disorganisation and collapse of arrayed sarcomeres. Animals expressing a full length translational fusion of gfp to myo-3 (myosin heavy chain A) were age synchronised at L1 stage and grown to young adulthood at 16°C (t = 0 h). Adult animals were then transferred to NGM RNAi plates [87] seeded with bacteria expressing dsRNA against genes indicated for a further 72 h to mid-adulthood. Displayed are sample images of defects in sarcomere structure observed for each indicated treatment. White arrow, minor sarcomere disorganisation (Examples: atn-1, deb-1); yellow arrow, major sarcomere disorganisation (Examples: unc-82, tln-1); red arrow, balled array of sarcomeres (Example: unc-112), red asterisk, torn array of sarcomeres (Example: unc-52). Scale bar represents 50 µm. Quantification of defects can be found in Figure 3; note that minor disorganisation (e.g. white arrows) was classed as normal for the purposes of quantification, whereas major disorganisation (e.g. yellow arrows) was classed as disorganised. (TIF) [file pgen.1002471.s001.tif]

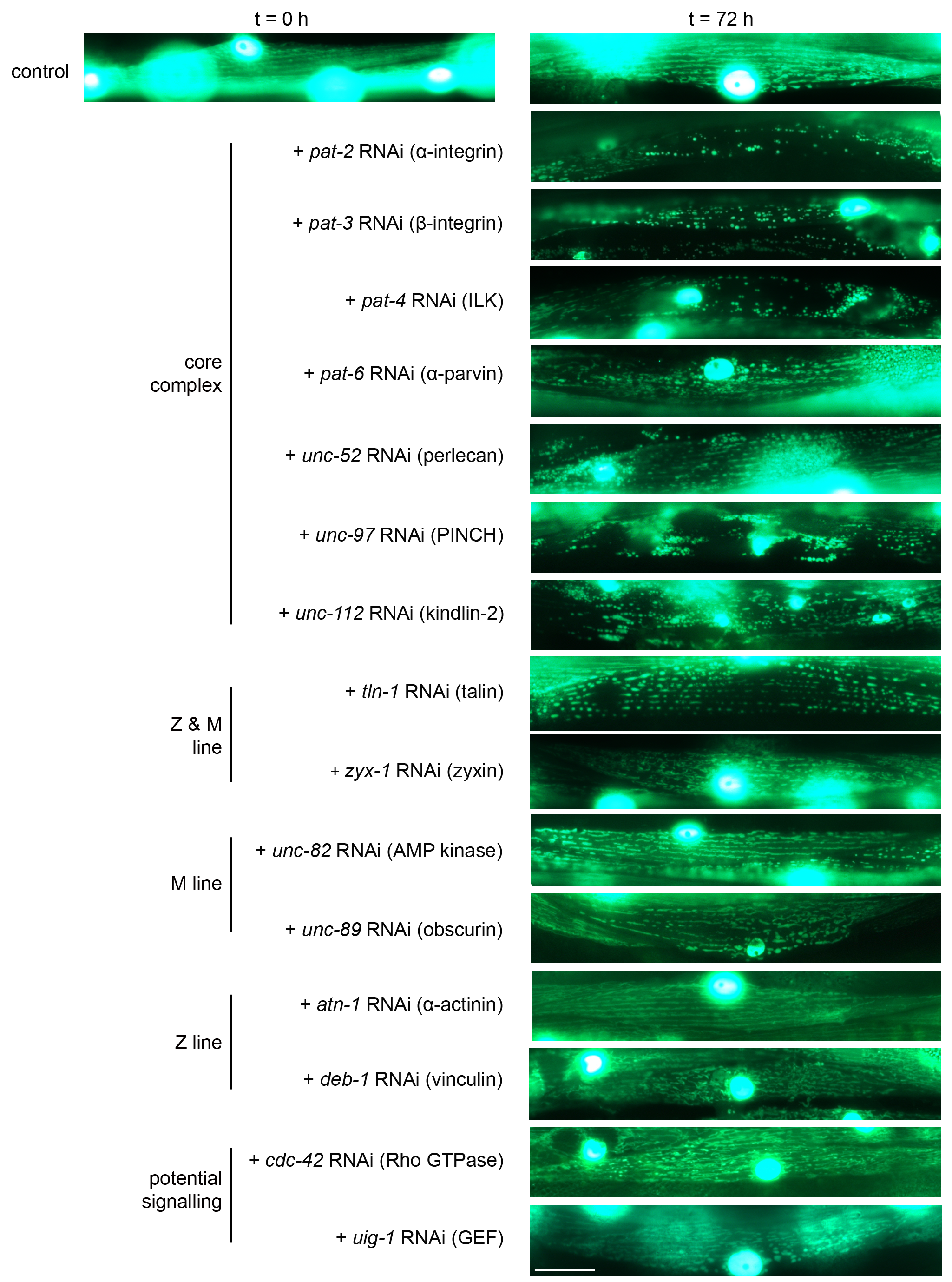

Supplement: Figure S2 — Acute loss of muscle attachment results in mitochondrial fragmentation. Animals expressing GFP-tagged mitochondria and nuclei were age synchronised at L1 stage and grown to young adulthood at 16°C (t = 0 h). Animals were then transferred to NGM RNAi plates [87] seeded with bacteria expressing dsRNA against genes indicated for an additional 72 h (mid-adulthood) at 20°C. Examples of networked, disorganised, and moderately and majorly fragmented mitochondria are displayed in Figure 4, where quantification of these defects can also be found. Minor fragmentation is usually classed as disorganised (for example the t = 72 h control image shows minor fragmentation on either side of the nucleus). Scale bar represents 50 µm. (TIF) [file pgen.1002471.s002.tif]

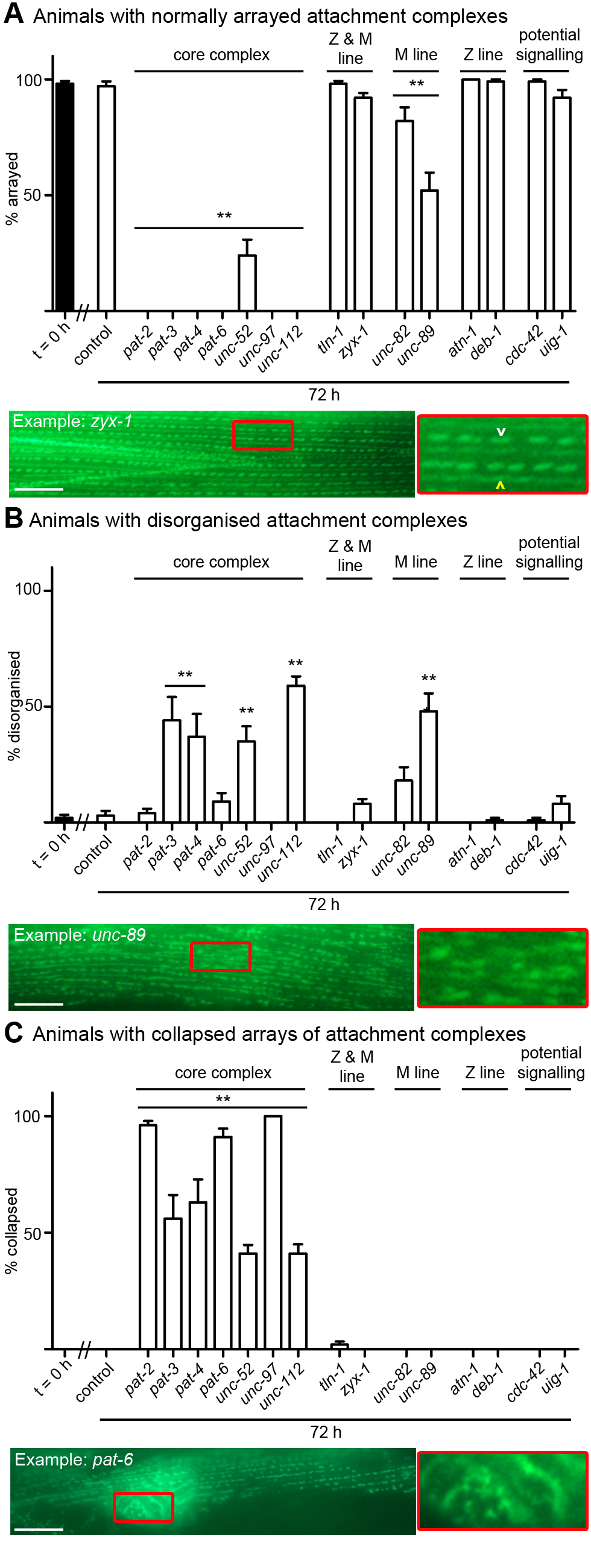

Supplement: Figure S3 — Acute genetic disruption of attachment complex genes results in disorganisation and collapse of attachment complexes. Animals expressing GFP-tagged attachment complexes (UNC-95::GFP) were age synchronised at L1 stage and grown to young adulthood at 16°C (t = 0 h). Animals were then transferred to NGM RNAi plates [87] seeded with bacteria expressing dsRNA against genes indicated for an additional 72 h (mid-adulthood) at 20°C. The 20 most Unc animals were picked and scored for identical defects in attachment complex structure in at least two muscles within the animal and this was repeated for 5 independent RNAi treatments (n = 100 animals per condition/time point). A) Percentage of animals where only normal arrays of attachment complexes were observed is displayed as average ± SEM. Below the graph is an example of an RNAi treated animal displaying normal arrays of attachment complexes (as indicated by straight parallel lines of GFP), these are enlarged 300% to the right of the micrograph. Note that Z-line attachment complexes, termed dense bodies, appear as punctate lines (white arrow) while M-line attachment complexes appear as a continuous line (yellow arrow). B) Percentage of animals where disorganisation of arrayed attachment complexes were observed is displayed as average ± SEM. Below the graph is an example of an RNAi treated animal displaying disorganised attachment complex arrays (as indicated by lack of straight parallel lines of GFP), these are enlarged 300% to the right of the micrograph. C) Percentage of animals where sarcomere arrays have collapsed into ball like structures is displayed as average ± SEM. Below the graph is an example of an RNAi treated animal displaying a collapsed array of sarcomeres (as indicated by GFP that appear as circular lines rather than straight lines, see also Figure 6C and 6D for examples), these are enlarged 300% to the right of the micrograph. **Significant difference from control t = 72 h, P<0.001 (two way repeated measures [file pgen.1002471.s003.tif]

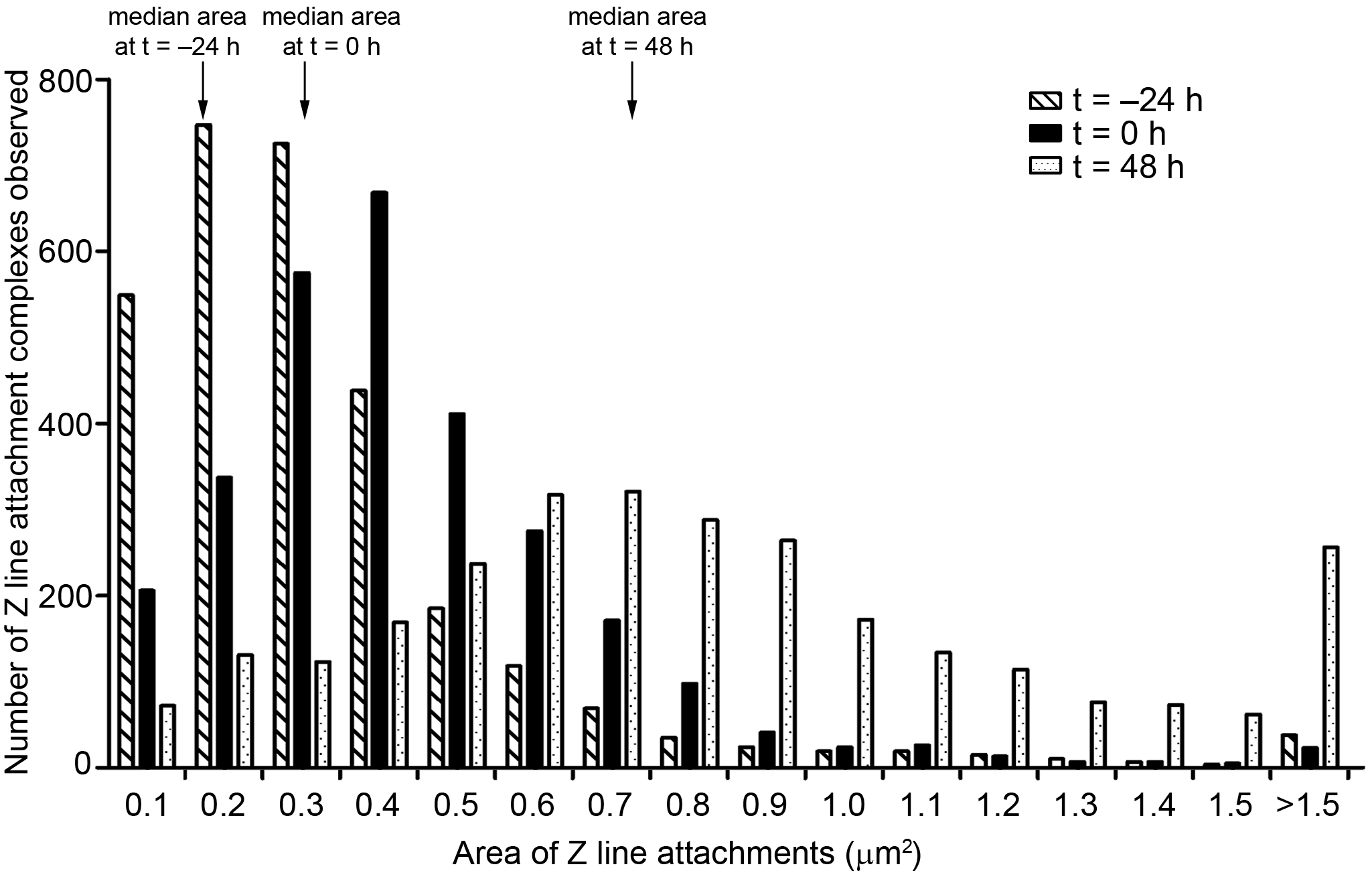

Supplement: Figure S4 — Z-line attachment complexes increase in area as C. elegans grow larger post-adulthood. Live worms of a strain carrying an unc-95::gfp fusion were examined by confocal microscopy at the fourth larval stage (t = −24 h, worm length = 0.75±0.02 mm), one day later as young adults (t = 0 h, length = 1.03±0.03 mm) or an additional two days later (t = 48 h, length = 1.44±0.02 mm). Images (7–9 worms of each group) were taken of a muscle cell in an approximately constant position (just posterior to the vulva, inner or outer cell in a band), fragments of adjacent cells and the M-lines removed manually in Photoshop, and the fluorescent Z-line attachment complexes (dense bodies) counted and their sizes measured using ImageJ software. The number of dense bodies per cell was t = −24 h, 333±26; t = 0 h, 412±24; t = 48 h, 402±31; we cannot state with high confidence whether or not the number of dense bodies per cell changes. The arrows indicate the medians of the dense body area distributions for t = −24 h (median area = 0.23 µm2, n = 3002), t = 0 h, young adults (median = 0.35 µm2, n = 2888) or t = 48 h (median = 0.71 µm2, n = 2813). In some rare instances, Image J fails to separate adjacent bodies, leading to an artifactually high size measurement (>1.5 µm2, at right); this distorts means but has little effect on medians. All pairwise comparisons show that differences between these size distributions are highly significant (P<0.0001, 2-tailed T-tests with unequal variances). (TIF) [file pgen.1002471.s004.tif]

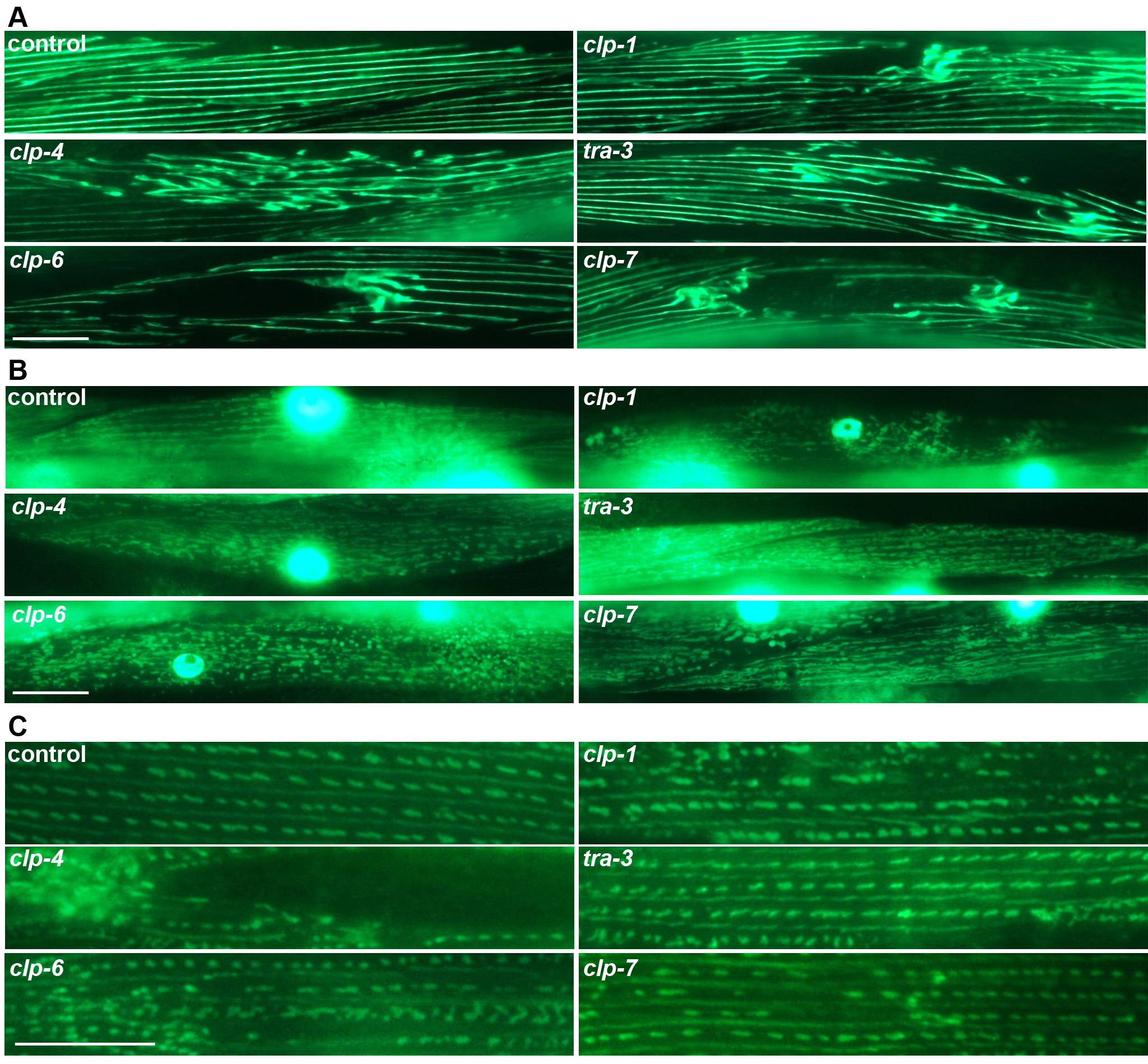

Supplement: Figure S5 — Calpains are important for maintenance of adult C. elegans muscle. A) Animals expressing a full length translational fusion of gfp to myo-3 (myosin heavy chain A) were age synchronised at L1 stage and grown to young adulthood at 16°C (t = 0 h). Adult animals were then transferred to NGM RNAi plates [87] seeded with bacteria expressing dsRNA against genes indicated for a further 72 h to mid-adulthood. B) Animals expressing GFP labelled mitochondria and nuclei were grown and treated as in A. C) Animals expressing GFP labelled attachment complexes (UNC-95::GFP) were grown, treated and analysed as in A. Quantification of defects can be found in Figure 8. Scale bars represent 15 µm. (TIF) [file pgen.1002471.s005.tif]

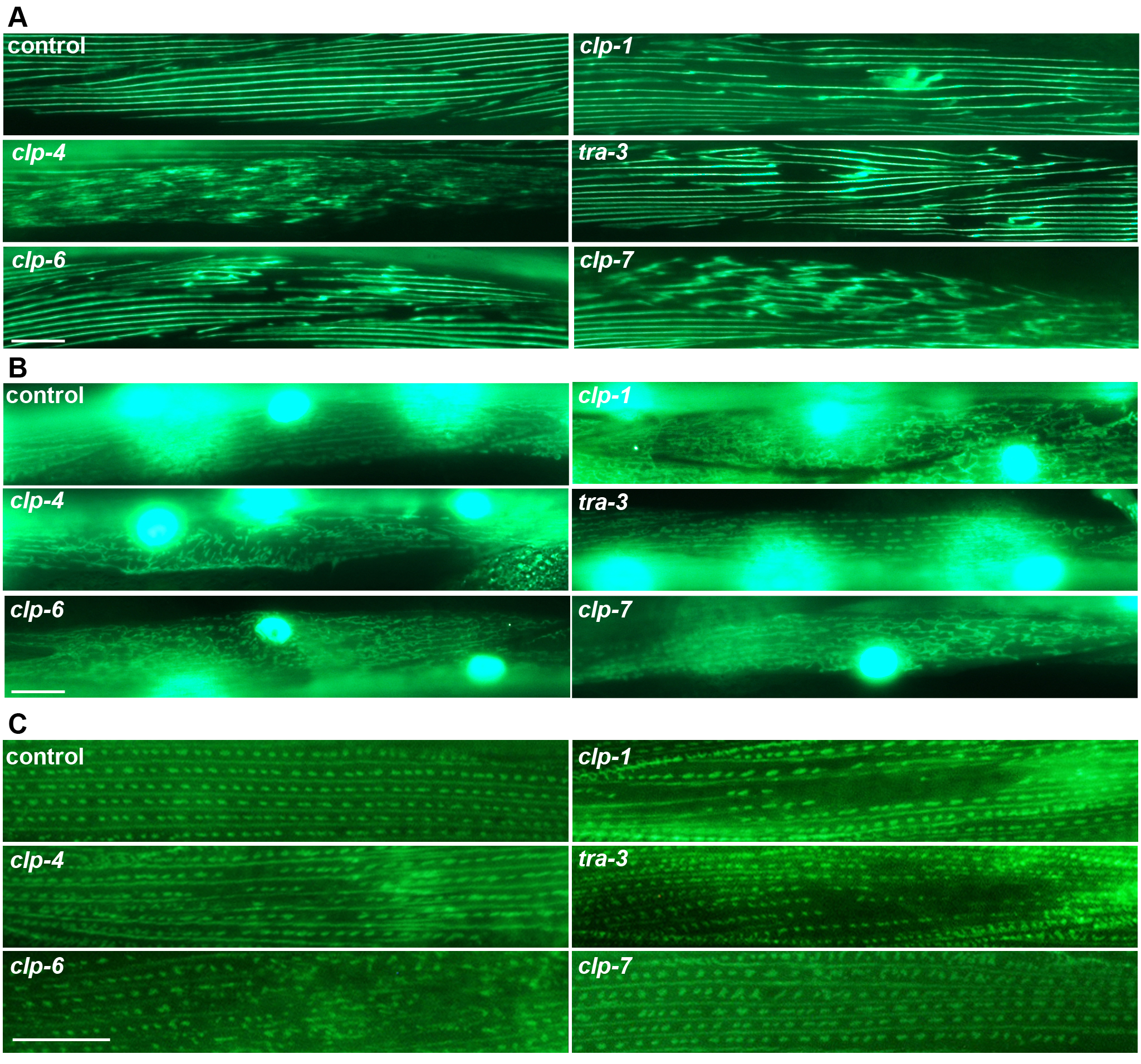

Supplement: Figure S6 — Calpains are important for development of C. elegans muscle. A) Animals expressing a full length translational fusion of gfp to myo-3 (myosin heavy chain A) were cultured from L4 stage to young adulthood under normal conditions at 20°C and on RNAi targeting clp-1, clp-4, tra-3, clp-6 or clp-7. B) Animals expressing GFP labelled mitochondria and nuclei were grown and treated as in A. C) Animals expressing GFP labelled attachment complexes (UNC-95::GFP) were grown, treated and analysed as in A. Quantification of defects can be found in Figure 9. Scale bars represent 15 µm. (TIF) [file pgen.1002471.s006.tif]
